# Effects of cilengitide derivatives on TGF-β1-induced epithelial-to-mesenchymal transition and invasion in gefitinib-resistant non-small cell lung cancer cells

**DOI:** 10.3389/fphar.2023.1277199

**Published:** 2023-10-20

**Authors:** Yujin Seo, Minji Seo, Jiyeon Kim

**Affiliations:** Department of Biomedical Laboratory Science, School of Health Science, Dankook University, Cheonan, Republic of Korea

**Keywords:** Cilengitide, cRGDwV, cRGDyV, EMT, NSCLC, A549GR

## Abstract

Long-term administration of tyrosine kinase inhibitors (TKIs) used for the treatment of non-small cell lung cancer (NSCLC) induces TKI resistance in cells. The appearance of resistant cells requires the combined administration of another therapeutic agent and may cause side effects in the gastrointestinal and central nervous system. In previous studies, we found that derivatives of cilengitide, a cyclic Arg-Gly-Asp (RGD) peptide, exert NSCLC apoptotic and anti–epithelial-mesenchymal transition (EMT) effects. In particular, cRGDwV and cRGDyV, which are cyclic peptides containing aromatic amino acids, were found to inhibit NSCLC cell growth, TGF-β1-induced EMT, and invasion. In this study, we confirmed the effects of cRGDwV and cRGDyV on proliferation, TGF-β1-induced EMT marker expression, migration, and invasion in gefitinib-resistant NSCLC A549 (A549GR) cells. In A549GR cells, cRGDwV and cRGDyV showed inhibitory effects on the expression of mesenchymal marker expression, migration, and invasion. These results indicate that cyclic RGD peptides containing aromatic amino acids can be used to inhibit mesenchymal marker expression as well as migration and invasion in gefitinib-resistant cells.

## 1 Introduction

The most common mutation seen in non-small cell lung cancer (NSCLC), accounting for approximately 85% of all lung cancer types, is in the tyrosine kinase domain of the epidermal growth factor receptor (EGFR) (Testa et al., 2018). A subset of NSCLC patients have active *EGFR* mutations, most of which are the deletion of exon 19 (Ex19del) and the L858R point mutation in exon 21 (Westover et al., 2018). In lung cancer cells, *EGFR* mutations are associated with anti-apoptotic signaling (e.g., through AKT and STAT), which promotes survival. In addition, there is a possibility of metastasis to the liver or central nervous system (CNS), so tyrosine kinase inhibitors (TKIs) targeting EGFR mutations are preferentially used as therapeutic agents (Sordella et al., 2004; Doebele et al., 2012; Shin et al., 2014; Iuchi et al., 2015). EGFR-TKIs were developed for the treatment of NSCLC and have been used through their first, second, and third generations to date (Westover et al., 2018; He et al., 2021).

Gefitinib, together with erlotinib and icotinib, is a first-generation TKI that reversibly binds to the ATP-binding domain of tyrosine kinase and inhibits EGFR downstream signaling, thereby exhibiting anticancer effects (Huang and Fu, 2015). Approved by the U.S. Food and Drug Administration (FDA) in 2015, gefitinib has been used for the first-line treatment of NSCLC patients with *EGFR* mutations, and many clinical studies have demonstrated that it prolongs progression-free survival when compared with existing treatment methods (Mok et al., 2009; Kazandjian et al., 2016). However, continuous use of TKIs for more than 9 months results in the disease not reaching full cure and spreading through the body due to resistance. Through many studies over the past few years, the mechanism of resistance has been investigated, and there has been continuous research on methods to increase treatment efficacy by suppressing the resistance mechanism (Mitsudomi et al., 2010; Sequist et al., 2011; Yu et al., 2013; Maemondo et al., 2020).

Peptides containing the Arg-Gly-Asp (RGD) sequence show high binding affinity for integrin α_v_β_3_ expressed in cancer cells, thereby reducing side effects and increasing the delivery rate of anticancer drugs to the body (Temming et al., 2005; Bianchini et al., 2019; Sartori et al., 2019). In particular, cilengitide [c (RGDf (*N*Me)V], a cyclic RGD peptide showing nanomolar activity and affinity for integrin receptors, has been used as a single or combined treatment for various diseases (Mas-Moruno et al., 2010). According to recent studies, the combined administration of these RGD sequence-containing peptides and existing anticancer agents is effective in inducing apoptosis and suppressing EMT and invasion by lung cancer cells (Park and Kim, 2020; Jeong and Kim, 2021; Jeong and Kim, 2022). In addition, we have demonstrated that combined treatment with the first-generation TKI gefitinib (or erlotinib) and the cyclic RGD peptide cilengitide and its derivatives have synergistic inhibitory effects on the growth of NSCLC cells and TGF-β1-induced EMT (Jeong and Kim, 2021; Jeong and Kim, 2022; Park et al., 2022; Seo and Kim, 2022).

EMT is a phenomenon caused by changes in the morphology of epithelial cells during processes such as embryogenesis, wound healing, and organ fibrosis, and is one of the main ways in which cancer cells acquire migratory and invasive properties, especially in the tumor microenvironment (Thiery et al., 2009; Siegel et al., 2011). EMT-triggered changes in cancer cells during the progression of lung cancer cause poor prognosis, such as increased mortality and metastasis to other organs, and thus can be an important target for lung cancer treatment. Transforming growth factor (TGF)-β is one of the major cytokines that induce EMT and is involved in cancer cell invasion and metastasis (Behrens et al., 1989; Buck and Knabbe, 2006; Barcellos-Hoff and Akhurst, 2009; Drabsch and ten Dijke, 2012). EMT induction by TGF-β occurs through the activation of signaling pathways such as Smad or non-Smad pathways including the Wnt/β-catenin and MAPK/ERK pathways (Heldin et al., 1997; Massagué, 1998; Massagué et al., 2005; Galliher et al., 2006; Medici et al., 2008; Chen et al., 2012). Therefore, inhibiting the signaling pathways associated with EMT and inducing the apoptosis of NSCLC cells can prevent changes in cell morphology that can affect metastasis and invasion.

In this study, we evaluated the anticancer and EMT inhibitory effects of the cilengitide derivatives cRGDwV and cRGDyV in gefitinib-resistant NSCLC cells. In gefitinib-resistant NSCLC A549 (A549GR) cells, cRGDwV and cRGDyV suppressed cell growth and inhibited TGF-β1-induced mesenchymal marker expression, migration, and invasion. In addition, we found that the inhibitory effects of cRGDwV and cRGDyV were increased when it was combined with gefitinib. These results suggest the use of cyclic RGD peptides can be an alternative means of reducing side effects that may occur when other types of TKIs are co-administered to patients with TKI-resistant NSCLC.

## 2 Materials and methods

### 2.1 Materials

Gefitinib (#SML1657) was purchased from Merck (United States), and cilengitide derivatives (cRGDwV and cRGDyV) were synthesized by Dr. Park (CHA Meditech, Korea) (Supplementary Figures S1–S17) (Park et al., 2022; Seo and Kim, 2022). Fetal bovine serum (FBS) was purchased from Thermo Fisher Scientific (United States). RPMI 1640 medium and antibiotics (100 U/mL penicillin and 100 μg/mL streptomycin) were purchased from Corning (United States). Recombinant human TGF-β1 was purchased from R&D Systems (United States). Antibodies against E-cadherin (#3195), α-smooth muscle actin (α-SMA) (#19245), p-ERK1/2 (#4370), ERK1/2 (#4695), and p-Smad2/3 (#8828) were purchased from Cell Signaling Technology (United States). Antibodies against Smad2/3 (#sc-133098), vimentin (#sc-6260), N-cadherin (#sc-59987), β-catenin (#sc-7199), and horseradish peroxidase (HRP)-conjugated secondary antibodies (#sc-516102 and #sc-2357), HRP-conjugated β-actin (#sc-47778), and HRP-conjugated GAPDH (#47724) were purchased from Santa Cruz Biotechnology (United States).

### 2.2 Cell line and determination of cell proliferation

Bovine pulmonary epithelium CPAE (KCLB No. 10209) and NSCLC A549 (KCLB No. 10185) cell lines were obtained from the Korean Cell Line Bank (Korea). CPAE and all NSCLC cells were maintained in RPMI 1640 medium containing 10% heat-inactivated FBS and 1% antibiotics (100 U/mL penicillin and 100 μg/mL streptomycin) and maintained at 37°C in a humidified atmosphere of 5% CO_2_. The gefitinib-resistant A549 (A549GR) cell line was established by treatment with a high concentration (2 μM) of gefitinib for at least 6 months (Supplementary Figure S18) (Shien et al., 2013).

Cell viability was detected using an EZ-Cytox Enhanced Cell Viability Assay Kit (DoGen, Korea). CPAE or A549GR cells were treated with gefitinib or peptides for 24–72 h in culture medium containing 10% FBS. The absorbance was measured by a Multiscan FC microplate photometer (Thermo Fisher Scientific, United States), and cell viability is presented as a percentage of control (untreated) cell viability.

### 2.3 Western blot analysis

A549GR cells were treated with TGF-β1 (5 ng/mL) or in combination with cRGDwV or cRGDyV. After a 48–72 h incubation, lysates of pooled cells were prepared using Nuclear and Cytoplasmic Extraction Reagent (Thermo Fisher Scientific, United States). The extracted protein was separated by SDS-PAGE and transferred to a nitrocellulose membrane, and the expression of each specific protein was detected with a primary antibody and an HRP-conjugated secondary antibody. The amount of each protein expressed was visualized using SuperSignal West Pico PLUS Chemiluminescent Substrate (Thermo Fisher Scientific, United States) and SuperSignal West Femto Maximum Sensitivity Substrate (Thermo Fisher Scientific, United States) and quantified using ImageJ software, and the relative signal ratio of each sample to the GAPDH or β-actin loading control is shown in the figures.

### 2.4 Analysis of combined drug effects

The effect of the combination of gefitinib with cRGDwV or cRGDyV was analyzed using the CalcuSyn software program (Biosoft, UK). To determine whether the outcome of combined treatment with two compounds was additive or synergistic, a combination index (CI) method derived from the median effect principle of Chou and Talalay was applied, and the CI was calculated with the formula published by Zhao et al. (Chou and Talalay, 1984; Zhao et al., 2004). In Supplementary Table S1, a CI of 1 indicates an additive effect between the two compounds, CI > 1 indicates antagonism, and CI < 1 indicates a synergistic effect.

### 2.5 Wound healing assay

A549GR cells were grown in 24-well plates. After 24-h serum starvation, cells were scratched using a SPLScar scratcher (SPL Life Sciences, Co., Ltd.), rinsed with PBS to remove debris, and incubated with TGF-β1 (5 ng/mL) and cRGDwV or cRGDyV in serum-free RPMI 1640 medium for 48 h. Images of migrated A549GR cells were captured at 0, 24, and 48 h. The wound healing index was expressed as a percentage and was calculated based on the length of three randomly selected zones at 0 h.

### 2.6 Invasion assay

A549GR cells were cultured in serum-free medium for 24 h and then treated with TGF-β1 (5 ng/mL) and cRGDwV or cRGDyV for 48 h. After harvesting, the cells were resuspended in serum-free medium for counting. Culture medium (30 μL) containing 5% FBS was added to the bottom of a Boyden chamber, and a gelatin-coated membrane filter, silicone gasket, and upper chamber were placed in this order, followed by cell suspension (5 × 10^3^ cells/50 μL) in the upper chamber and incubation at 37°C in 5% CO_2_. After 4 h of incubation, cells that had penetrated the membrane were stained using the Differential Quick III stain kit (Polysciences, United States).

### 2.7 Statistical analysis

All experiments were conducted in triplicate (*n* = 3), and representative figures of the results of at least three independent experiments are shown. Data are presented as the mean ± SD. Significant differences were evaluated by one-way ANOVA with post-hoc Tukey HSD test using Microsoft Excel. *p* < 0.05 was considered significant (* or ^#^).

## 3 Results

### 3.1 Effect of cRGDwV and cRGDyV on cell viability in A549GR cells

**FIGURE 1:**
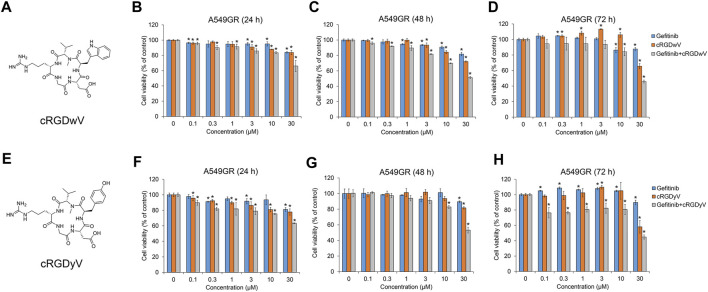
Combination effect of gefitinib with cilengitide derivatives on gefitinib-resistant A549 (A549GR) cell proliferation. **(A,E)** Chemical structures of cRGDwV **(A)** and cRGDyV **(E)**. **(B–D)** A549GR cells were treated with gefitinib or cRGDwV or gefitinib with cRGDwV for 24–72 h **(F–H)** A549GR cells were treated with gefitinib or cRGDyV or gefitinib with cRGDyV for 24–72 h. After incubation, cell viability was measured by the CCK-8 assay. Experiments were performed in triplicate. Data are the mean ± SD.

Among the seven cilengitide derivatives synthesized in previous studies, cRGDwV (R-1) and cRGDyV (R-7) showed strong inhibitory effects on NSCLC cell growth without significant cytotoxicity to normal lung endothelial cells (Park et al., 2022; Seo and Kim, 2022). In addition, it was confirmed that these peptides exhibit a synergistic effect in inducing NSCLC cell death when co-administered with gefitinib (Park et al., 2022; Seo and Kim, 2022). These two derivatives did not show significant cytotoxicity as measured by the growth of normal bovine lung epithelium CPAE cells (Park et al., 2022; Seo and Kim, 2022). On the basis of these results, we confirmed the effect of co-treatment with gefitinib and cRGDwV or cRGDyV on gefitinib-resistant A549 (A549GR) cells. As shown in Figure 1, A549GR cells were not significantly affected by gefitinib treatment alone, but A549GR cell growth was suppressed when gefitinib was used in combination with cRGDwV (Figures 1A–D) or cRGDyV (Figures 1E–H). The combination index (CI) was calculated using the raw data in Figure 1 (Supplementary Table S1). At a concentration of 0.3 μM or higher, all CI values were less than 1. This result means that cell death can be induced more effectively in gefitinib-resistant cells when treated with either cRGDwV or cRGDyV together with gefitinib. The combination treatment of gefitinib and cRGDwV or cRGDyV did not significantly affect the growth of CPAE cells (Supplementary Figures S19, S20). This result indicates that cRGDwV and cRGDyV can increase the growth inhibitory effect of gefitinib on gefitinib-resistant cells without significant cytotoxicity to normal lung endothelial cells.

### 3.2 Effects of cRGDwV and cRGDyV on the expression of EMT markers and related signaling in A549GR cells

TGF-β1 treatment decreases the expression of the epithelial marker E-cadherin, but increases the expression of mesenchymal markers such as N-cadherin, vimentin, and α-SMA (Kalluri and Weinberg, 2009; Jakobsen et al., 2016). We have confirmed in previous studies that cRGDwV (R-1) and cRGDyV (R-7) inhibit the expression of N-cadherin, vimentin, and α-smooth muscle actin (SMA), which are TGF-β1-induced mesenchymal markers (Park et al., 2022; Seo and Kim, 2022). Those studies also confirmed that mesenchymal marker expression and related signaling processes are suppressed when wild-type A549 cells are treated with gefitinib.

**FIGURE 2:**
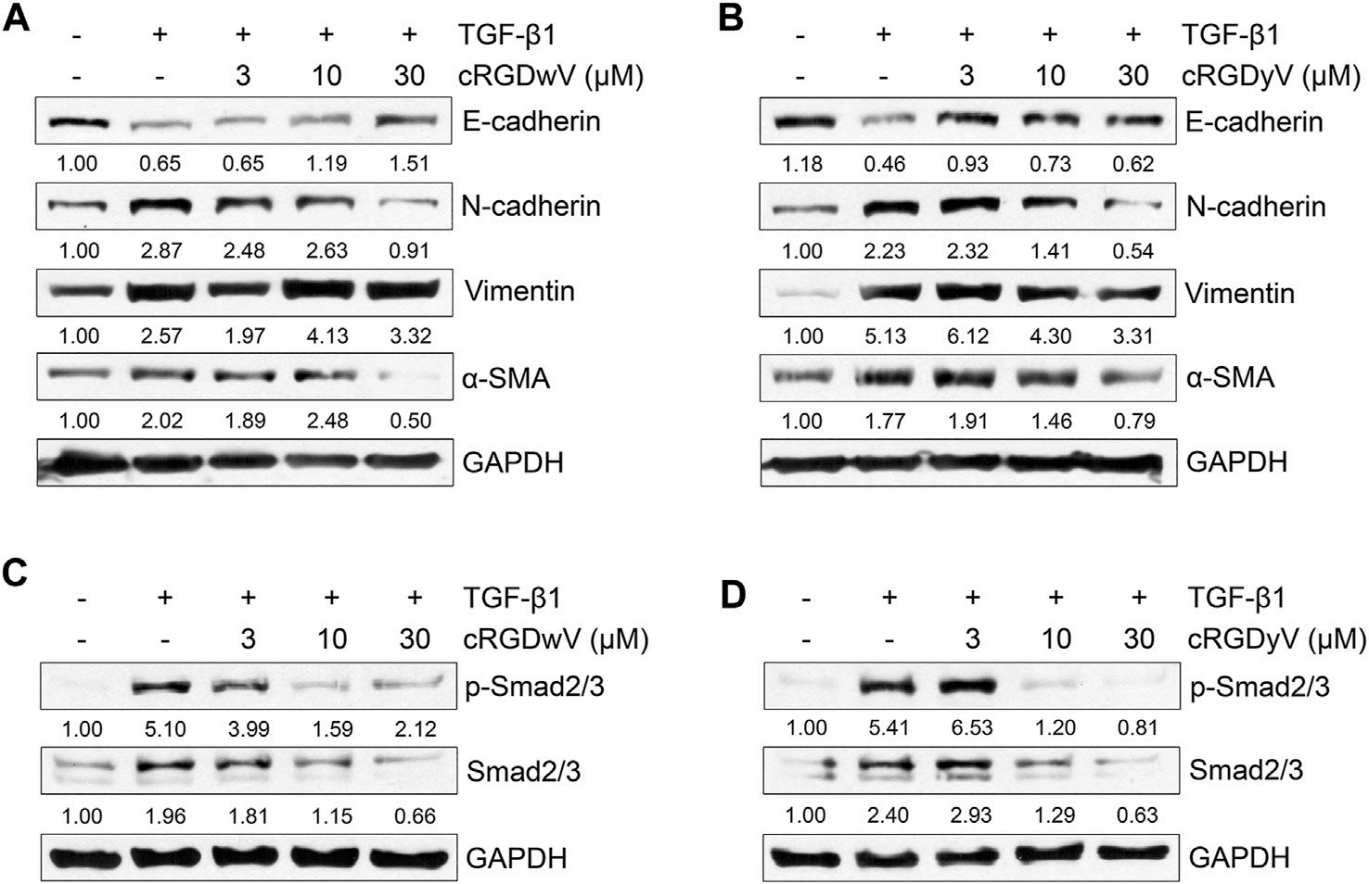
Effect of cilengitide derivatives on TGF-β1-induced EMT marker expression and related signaling pathways in A549GR cells. Serum-deprived A549GR cells were treated with TGF-β1 (5 ng/mL) and cRGDwV **(A,C)** or cRGDyV **(B,D)** for 72 h. Expression of EMT markers and phosphorylation of nuclear Smad2/3 was determined by western blot analysis. GAPDH was used as a loading control.

To verify the effects of cRGDwV and cRGDyV on gefitinib-resistant A549 cells, the expression of TGF-β1-induced EMT markers was confirmed in A549GR cells. As shown in Figures 2A, B, the expression of E-cadherin suppressed by TGF-β1 was restored by cRGDwV and cRGDyV, and the increases in N-cadherin and α-SMA expression were suppressed. Although not as strongly inhibited as N-cadherin expression, at 30 μM peptide vimentin expression was also inhibited.

To verify the inhibitory effects of cRGDwV and cRGDyV on TGF-β1-induced mesenchymal marker expression, we also confirmed the phosphorylation of Smad2/3, a key mediator in the EMT-related signaling pathway, and the expression of non-Smad signaling-related proteins. Recent studies have reported that cRGDwV or cRGDyV alone inhibits Smad-dependent or Smad-independent signaling processes in gefitinib-sensitive A549 cells, thereby affecting EMT phenotype change (Park et al., 2022; Seo and Kim, 2022). As shown in Figures 2C, D, treatment with cRGDwV or cRGDyV inhibits TGF-β1-induced phosphorylation of nuclear Smad2/3 in a dose-dependent manner.

In addition, we checked the phosphorylation of ERK1/2 and the expression of β-catenin to confirm the effects of cRGDwV and cRGDyV on non-Smad signaling. In Smad-independent signaling, cRGDwV or cRGDyV did not significantly inhibit phosphorylation of ERK1/2 and nuclear translocation of β-catenin in A549GR cells (Supplementary Figure S21). These results indicate that Smad-dependent signaling is maintained more strongly in gefitinib-resistant cells than in cells that are not resistant, and that the combined administration of gefitinib with either one of two cilengitide derivatives may inhibit EMT by inhibiting Smad-dependent signaling.

### 3.3 Effect of cRGDwV and cRGDyV on migration and invasion in A549GR cells

**FIGURE 3:**
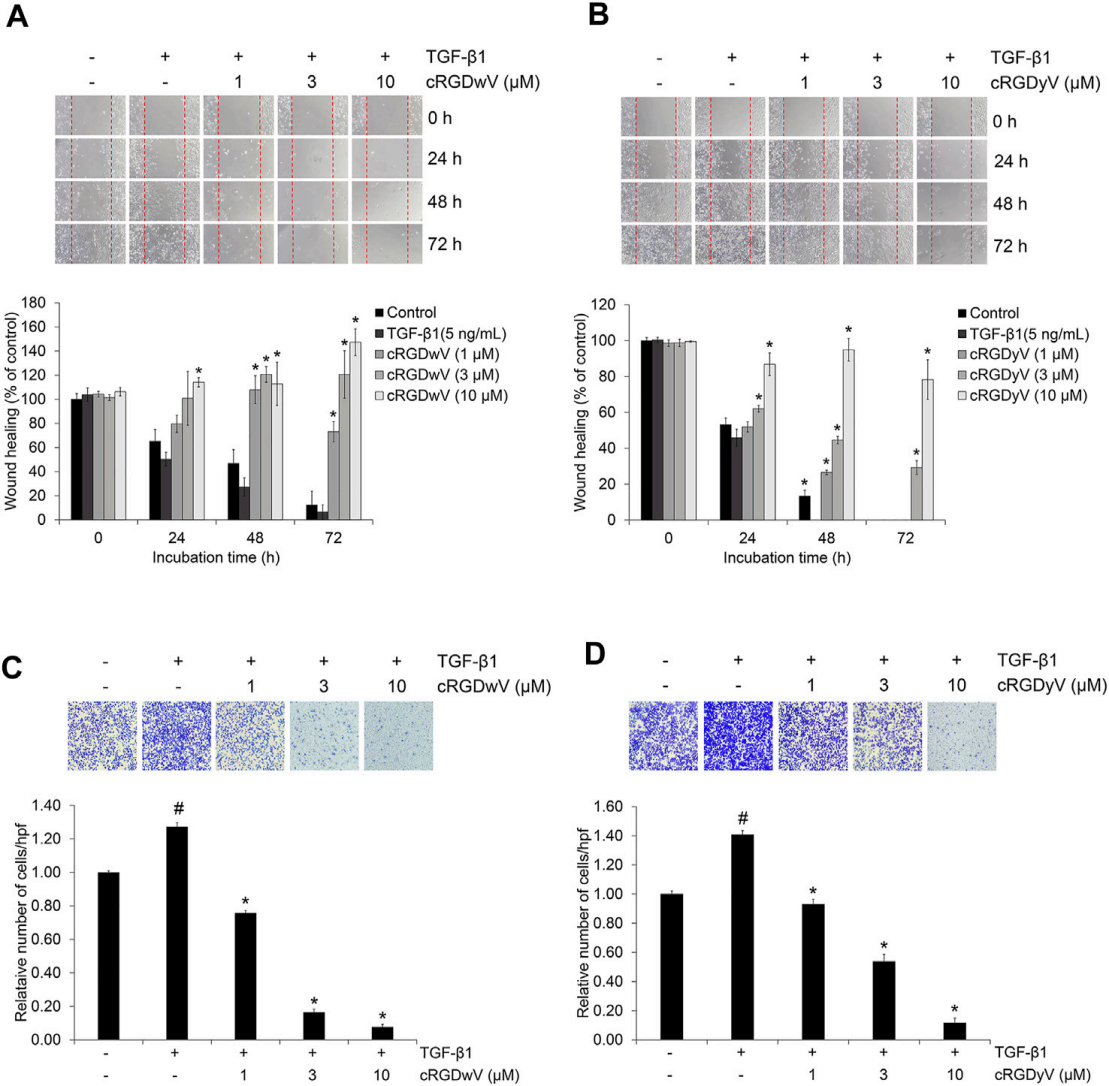
Effect of cilengitide derivatives on TGF-β1-induced migration and invasion in A549GR cells. Serum-deprived A549GR cells were scratched and treated with TGF-β1 (5 ng/mL) and cRGDwV **(A,C)** or cRGDyV **(B,D)** for 72 h. Images of A549GR cells that had migrated were captured at 0, 24, and 48 h. The wound healing index is expressed as a percentage and was calculated based on the length of three randomly selected zones at 0 h ^#^ *p* < 0.05 *versus* control; *p < 0.05 *versus* the group treated with TGF-β1 only.

EMT in cancer cells plays a very important role in metastasis by promoting migration and invasion (Mittal, 2018). In recent studies on A549 cells, we confirmed the inhibitory effect of combined treatment with gefitinib and either cilengitide derivative cRGDwV or cRGDyV on TGF-β1-induced invasion across a gelatin-coated membrane (Seo and Kim, 2022). The combined treatment of gefitinib and cRGDwV or cRGDyV inhibited invasion more effectively than gefitinib treatment alone. Based on these previous studies, we also investigated inhibitory effects of cRGDwV and cRGDyV on A549GR cell migration and invasion. As shown in Figures 3A, B, TGF-β1-treated A549GR cells in the scratched monolayer showed a pattern of gathering together over time, and all scratches were filled after 72 h. However, in the cRGDwV- or cRGDyV-treated group, scratches were not filled, and they persisted. This result was similar to those of the invasion assay. Treatment with TGF-β1 induced A549GR cells to cross the gelatin-coated membrane, but the number of infiltrating cells was reduced (Figures 3C, D). These results show that cRGDwV and cRGDyV alone are excellent inhibitors of migration and invasion, and demonstrate the possibility that they can act synergistically when used together with drugs showing anticancer effects in NSCLC.

### 3.4 Combination effect of gefitinib with cRGDwV and cRGDyV on the expression of EMT markers, related signaling and invasion in A549GR cells

**FIGURE 4:**
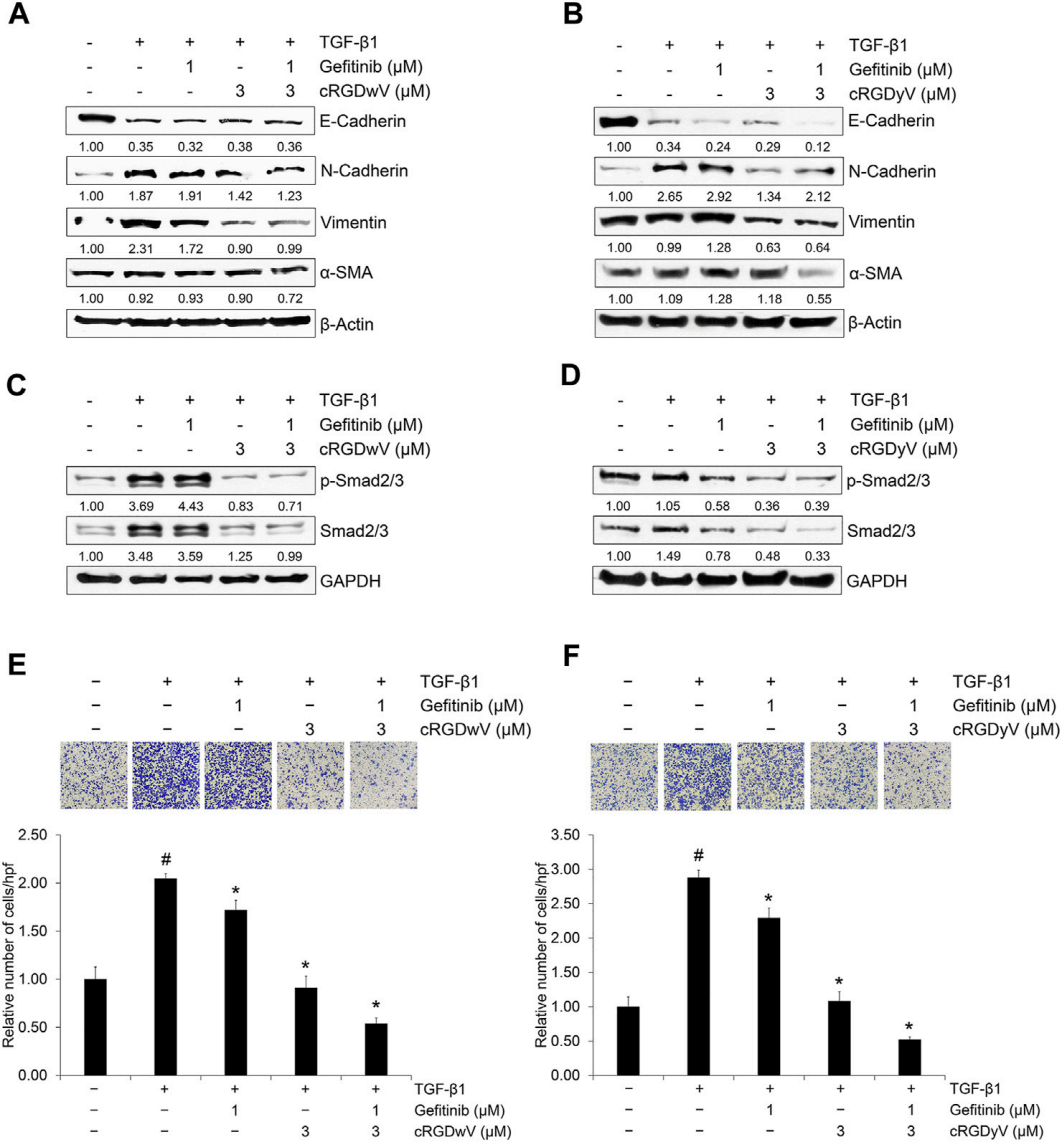
Combination effect of gefitinib with cilengitide derivatives on TGF-β1-induced expression of EMT markers, related signaling and invasion in A549GR cells. **(A–F)** Serum-deprived A549GR cells were treated with TGF-β1 (5 ng/mL) or gefitinib (1 μM) or in combination with cRGDwV (3 μM) **(A,C,E)** or cRGDyV (3 μM) **(B,D,F)** for 72 h **(A–D)** Expression of EMT markers and nuclear expression of phosphorylated Smad2/3 and endogenous Smad2/3 was determined by western blot analysis. β-actin was used as a loading control. **(E,F)** The number of invading cells was evaluated using a Boyden chamber assay. The number of cells that invaded the gelatin-coated membrane is represented by an average number of cells per three randomly selected high-power fields (HPFs). The detailed experimental procedure is described in the Materials and Methods section. ^#^ *p* < 0.05 *versus* control; *p < 0.05 *versus* the group treated with TGF-β1 only.

We confirmed the combined effects of cRGDwV and cRGDyV with gefitinib on the expression of EMT markers and invasion in A549GR cells. As shown in Figures 4A, B, treatment with gefitinib (1 μM) or cRGDwV (3 μM) or cRGDyV (3 μM) alone did not significantly inhibit mesenchymal marker expression, whereas treatment with cRGDwV or cRGDyV inhibited the expression of mesenchymal makers such as N-cadherin, vimentin, and α-SMA. In particular, cRGDwV was effective in suppressing the expression of N-cadherin and vimentin, and cRGDyV seemed to help suppress the expression of α-SMA. This inhibitory effect of mesenchymal marker expression following the combined administration is thought to originate from inhibition of phosphorylation of Smad2/3 (Figures 4C, D), and the non-Smad signaling pathway was not suppressed (Supplementary Figure S22).

In addition, the invasion-inhibitory effect of low-dose gefitinib (1 μM) was not significant in A549GR cells, whereas cRGDwV and cRGDyV alone or in combination with gefitinib inhibited invasion significantly (Figures 4E, F). These results suggest that the effect of combined administration of gefitinib with cyclic peptides such as cRGDwV and cRGDyV may be more effective than treatment with high concentrations of gefitinib to inhibit the migration and invasion of NSCLC cells resistant to gefitinib.

## 4 Discussion

We have already confirmed in previous studies that first-generation TKIs such as gefitinib or erlotinib inhibit the growth of NSCLC and are effective in inhibiting TGF-β1-induced EMT and invasion. In addition, a synergistic effect was confirmed when cyclic peptides such as a cilengitide were used in combination treatment (Jeong and Kim, 2021; Jeong and Kim, 2022; Park et al., 2022; Seo and Kim, 2022). RTK inhibitors such as erlotinib and gefitinib are used to treat NSCLC but have limitations because the long-term use of them alone is the main cause of the emergence of resistant cells due to compensatory activation of intracellular signaling mechanisms (Sequist et al., 2011; Yu et al., 2013; Huang and Fu, 2015). In the process of acquiring EGFR-TKI resistance, cells are known to undergo changes such as loss of *EGFR* mutation, secondary EGFR *T790M* and minor mutations, *MET* amplification, and the activation of the MET/HGF axis and EMT (Huang and Fu, 2015; He et al., 2021). Third-generation EGFR-TKIs such as abivertinib, rociletinib, nazartinib, and olmutinib, which target the EGFR *T790M* mutation, can be used to overcome resistance caused by first- and second-generation EGFR-TKIs used as first-line treatment for NSCLC. However, in the case of long-term use, resistance to third-generation EGFR-TKIs cannot be avoided, and the mechanism of resistance is very complex and incompletely understood (He et al., 2021).

In order to overcome the resistance caused by the use of TKIs, a new chemotherapy that can suppress the proliferation and metastasis of NSCLC is needed, rather than one co-administered with TKIs that can cause resistance again. Thus, in recent studies, we confirmed the inhibitory effects on NSCLC cell growth and EMT of the first-generation TKIs gefitinib and erlotinib and the RGD-based cyclic peptide cilengitide (Jeong and Kim, 2021; Jeong and Kim, 2022). Cilengitide induced cell death in NSCLC by itself, but showed a synergistic effect on cell growth inhibition when combined with gefitinib or erlotinib. It was also found to be effective in suppressing mesenchymal marker expression and related signaling processes induced by TGF-β1 (Jeong and Kim, 2021; Jeong and Kim, 2022). Subsequent studies confirmed the effects of cilengitide derivatives: the peptides cRGDwV and cRGDyV containing aromatic amino acids inhibited NSCLC cell growth and EMT (Park et al., 2022; Seo and Kim, 2022). However, in NSCLC chemotherapy using TKIs, it was considered necessary to evaluate the effects of the peptides on TKI-resistant cells because it is expected that TKI resistance will eventually appear in NSCLC cells even with long-term peptide co-administration.

To confirm whether the cilengitide derivatives cRGDwV and cRGDyV have anticancer effects in TKI-resistant NSCLC, we established A549 cells resistant to gefitinib, a first-generation TKI, and used them to confirm the effects of the cilengitide derivatives. The experiments demonstrated that gefitinib-resistant A549 (A549GR) cells showed less gefitinib-induced cell death than did wild-type A549 cells, and high phosphorylation of Smad2/3, which is related to EMT. Since gefitinib alone does not show a sufficient anticancer effect in gefitinib-resistant cells, the result was confirmed by co-administration of cilengitide derivatives, which were shown to induce cell death and inhibit EMT in previous studies (Park et al., 2022; Seo and Kim, 2022). In A549GR cells, the cilengitide derivatives cRGDwV and cRGDyV had cell death-inducing effects similar to those of gefitinib, but a synergistic effect was observed when either derivative and gefitinib were administered together. In addition, regarding the expression of EMT markers induced by TGF-β1, when administered at the concentration that inhibits mesenchymal marker expression in gefitinib-sensitive A549 cells, gefitinib did not inhibit mesenchymal marker expression significantly in A549GR cells, and its inhibitory effect in combination with cRGDwV or cRGDyV was confirmed (Jeong and Kim, 2021). This inhibition was judged to affect migration and invasion processes associated with EMT. Mesenchymal marker expression was not significantly inhibited in A549GR cells when treated with cRGDwV or cRGDyV alone, but migration and invasion were effectively inhibited as the concentration increased, and a synergistic effect was observed when cells were co-treated with gefitinib. As in the previous study, we expected that cilengitide derivatives would suppress the expression of TGF-β1-induced mesenchymal makers in A549GR cells, and that this effect would also affect migration and invasion. However, genetic or other changes in A549GR cells are presumed to interfere with the EMT inhibitory effect of cRGDwV and cRGDyV. Although the anti-cancer effect of these cilengitide derivatives is somewhat insufficient in inhibiting EMT, it has been confirmed that the effect of inhibiting migration and invasion is good, so a greater anti-cancer effect can be expected when they are administered in combination with second- or third-generation lung cancer drugs.

We plan to continue to study the establishment of NSCLC cells resistant to second- and third-generation TKIs and the anti-cancer effects of cilengitide derivatives by extending the results of this work on A549GR cells. In addition, we will aim to develop peptides that act as antagonists of integrin receptors and that help therapeutic agents such as TKIs enter cancer cells more easily by designing and synthesizing new cyclic peptides containing aromatic amino acids. Peptide therapeutics are continuously being studied as an alternative approach to reducing the side effects of small molecule compound-based drugs, but they take a lot of time and money to develop due to *in vivo* degradation and low stability. For this reason, many pharmaceutical companies are applying existing safety-tested drugs to new diseases before developing new drugs, in what is called “drug repositioning” (Chong and Sullivan, 2007; Hanusova et al., 2015). Therefore, in NSCLC, the combined administration of conventional therapeutic agents such as TKIs and RGD-based cyclic peptides showing anti-cancer effects is expected to provide a new treatment method that can reduce side effects and overcome resistance. It is still necessary to verify their effects in other TKI-resistant cells and the effect of co-administering these peptides with second- and third-generation TKIs, and to conduct additional studies such as animal experiments, but the results of this study provide a new clue to the development of NSCLC treatments using peptides.

## Data Availability

The original contributions presented in the study are included in the article/Supplementary Material, further inquiries can be directed to the corresponding author.
