# Supplementary material for: Effects of cilengitide derivatives on TGF-β1-induced epithelial-to-mesenchymal transition and invasion in gefitinib-resistant non-small cell lung cancer cells
Source: Front Pharmacol. 2023 Oct 20;14:1277199. doi: 10.3389/fphar.2023.1277199 (PMC10622769; doi:10.3389/fphar.2023.1277199)

Fig. 2A

A549GR_R-1(cRGDwV)_72 h_cytosol

1. control 2. TGF-β1 (5ng / ml) 3. TGF-β1 + R-1 (cRGDwV) 3 μM

4. TGF-β1 + R-1 10 μM 5. TGF-β1 + R-1 30 μM

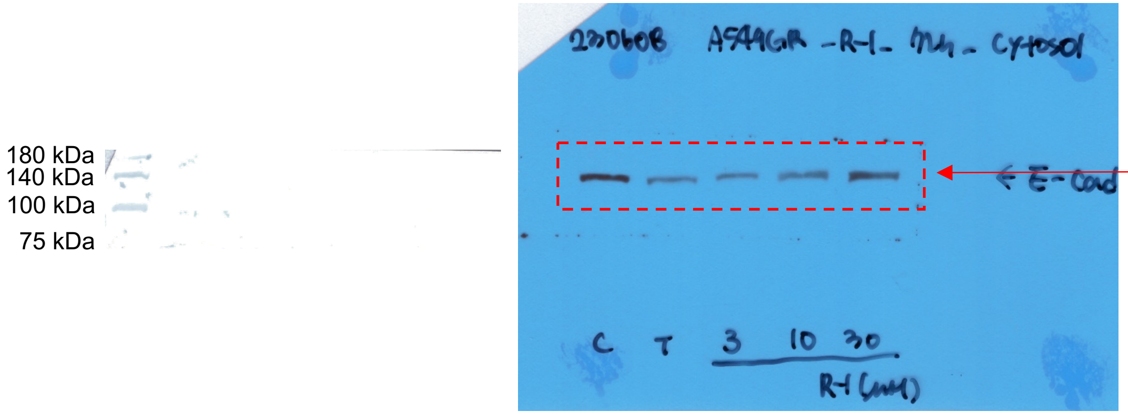

E-cadherin

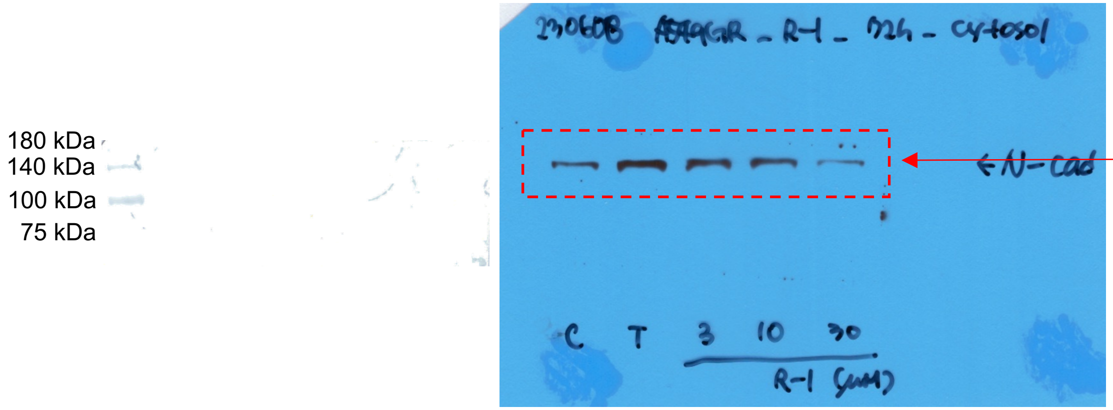

N-cadherin

Fig. 2A

A549GR_R-1(cRGDwV)_72 h_cytosol

1. control 2. TGF-β1 (5ng/ml) 3. TGF-β1 + R-1 (cRGDwV) 3 μM

4. TGF-β1 + R-1 10 μM 5. TGF-β1 + R-1 30 μM

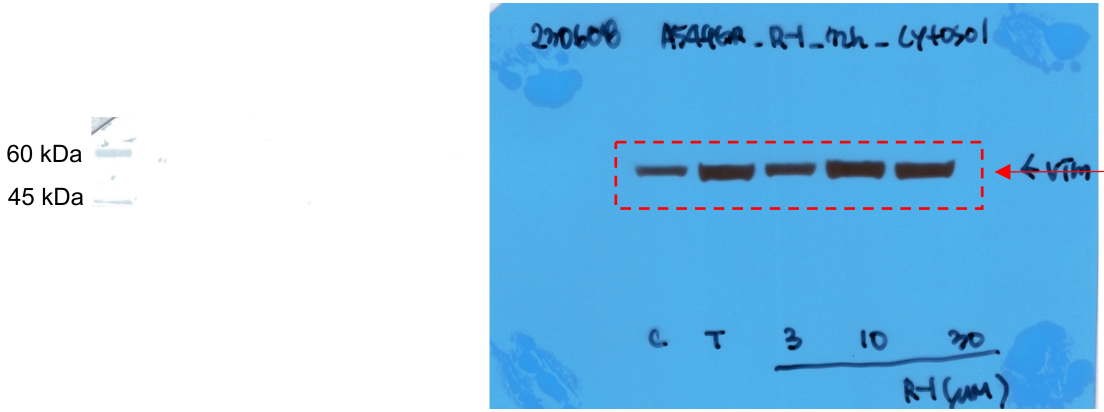

Vimentin

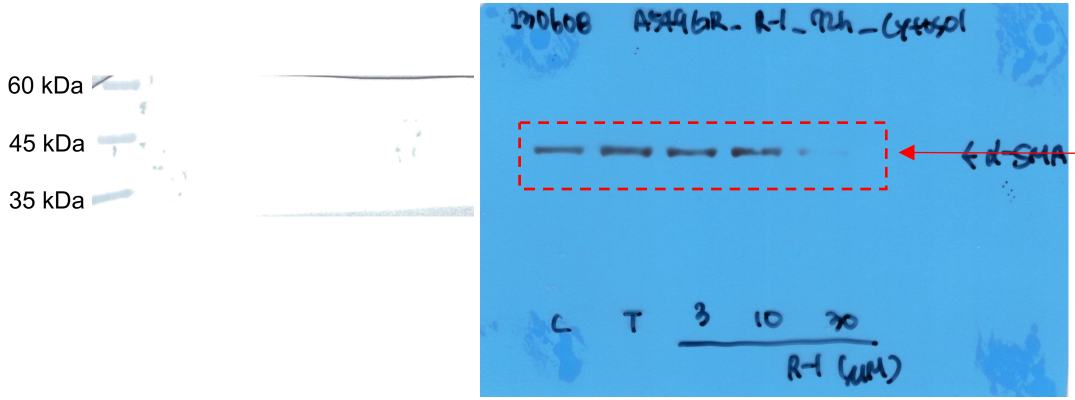

α-SMA

Fig. 2A

A549GR_R-1(cRGDwV)_72 h_cytosol

1. control 2. TGF-β1 (5ng/ ml) 3. TGF-β1 + R-1 (cRGDwV) 3 μM

4. TGF-β1 + R-1 10 μM 5. TGF-β1 + R-1 30 μM

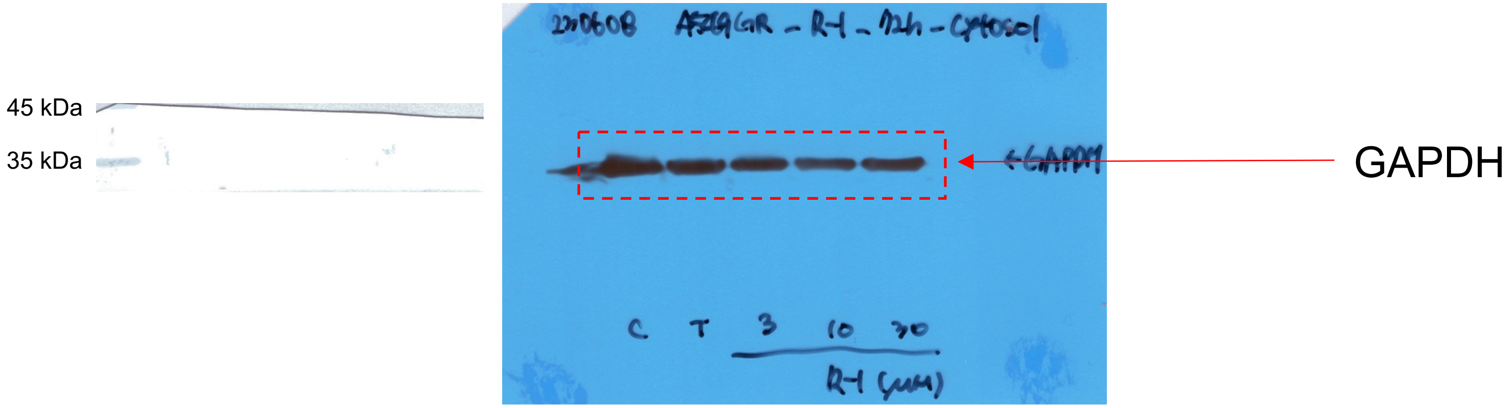

Fig. 2B

A549GR_R-7(cRGDyV)_72 h_cytosol

1. control 2. TGF-β1 (5ng/ml) 3. TGF-β1 + R-7 (cRGDyV) 3μM

4. TGF-β1 + R-7 10μM 5. TGF-β1 + R-7 30μM

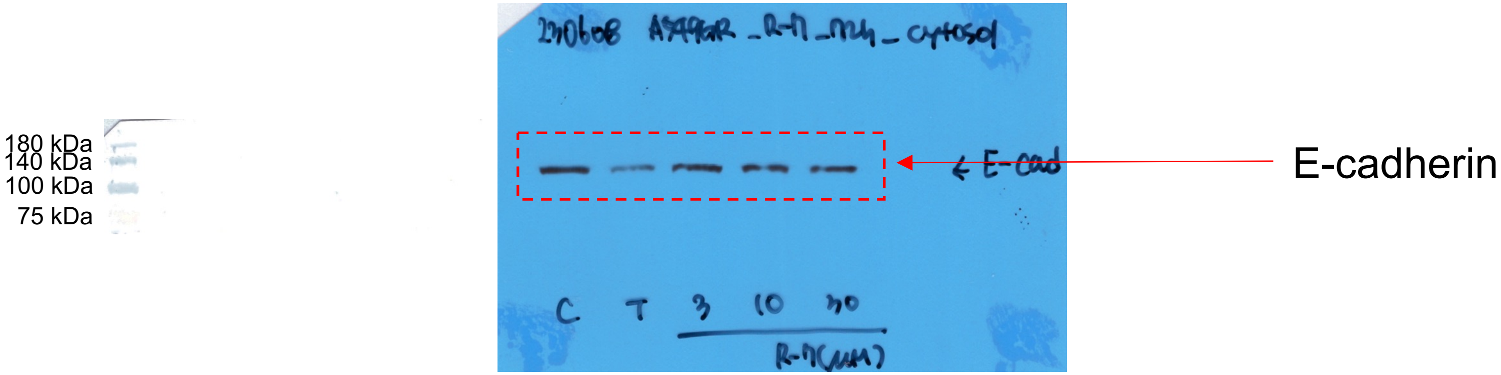

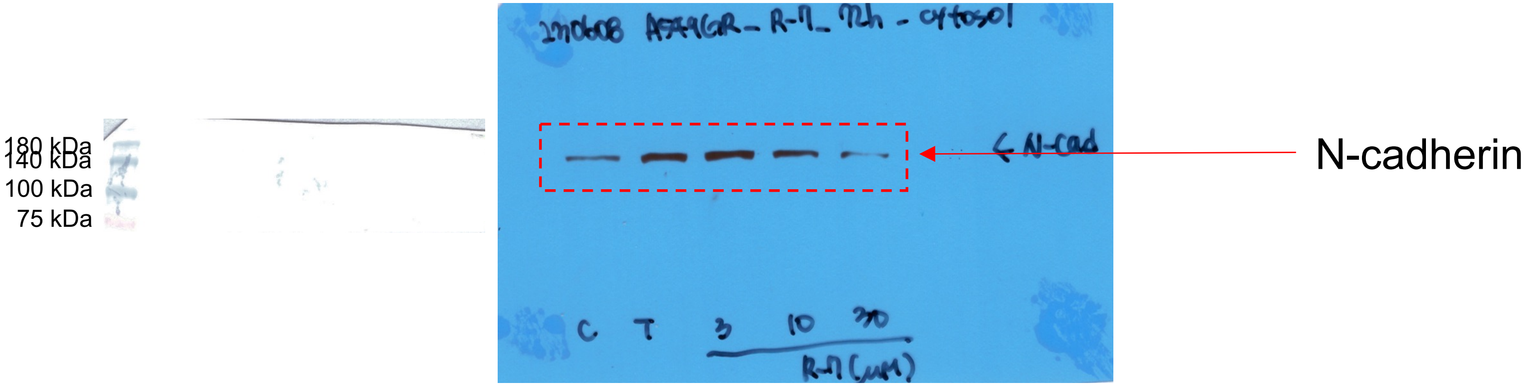

Fig. 2B

A549GR_R-7(cRGDyV)_72 h_cytosol

1. Control
2. TGF-β1 (5ng/mℓ)
3. TGF-β1 + R-7 (cRGDyV) 3μM
4. TGF-β1 + R-7 10μM
5. TGF-β1 + R-7 30μM

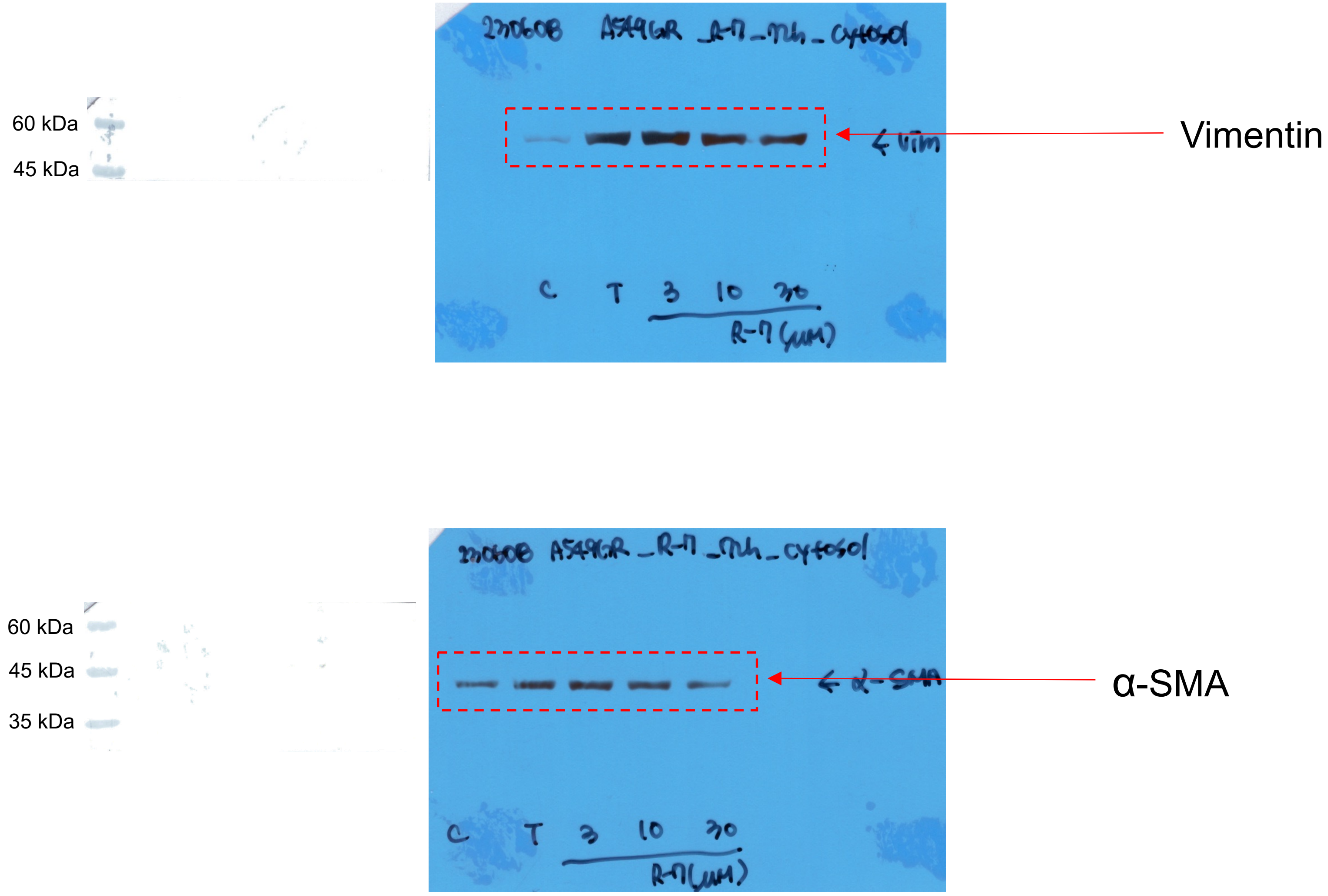

Fig. 2B

A549GR_R-7(cRGDyV)_72 h_cytosol

1. control
2. TGF-β1 (5ng/ml)
3. TGF-β1 + R-7 (cRGDyV) 3μM
4. TGF-β1 + R-7 10μM
5. TGF-β1 + R-7 30μM

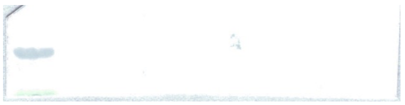

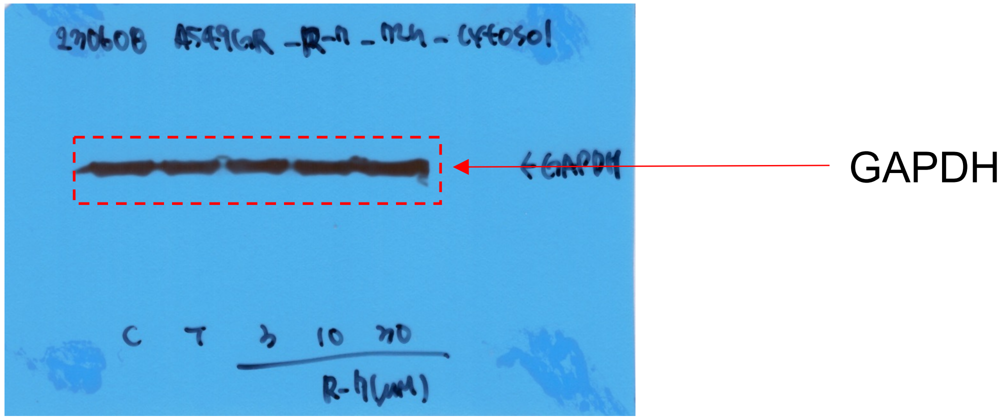

GAPDH

Fig. 2E

A549GR_R-1(cRGDwV)_72 h_nucleus

1. Control 2. TGF-β1 (5ng/ml) 3. TGF-β1 + R-1 3 μM

4. TGF-β1 + R-1 10 μM 5. TGF-β1 + R-1 30 μM

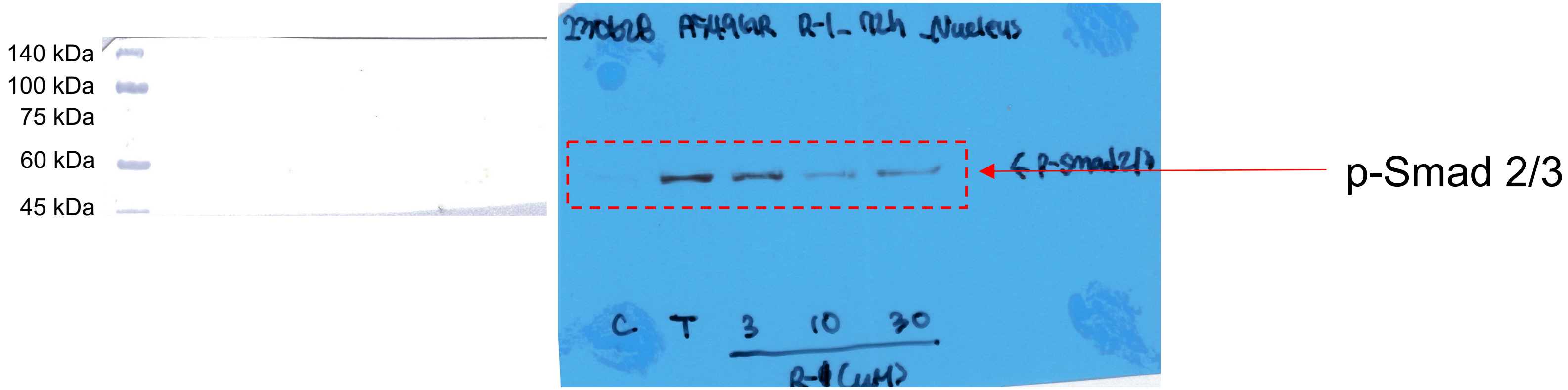

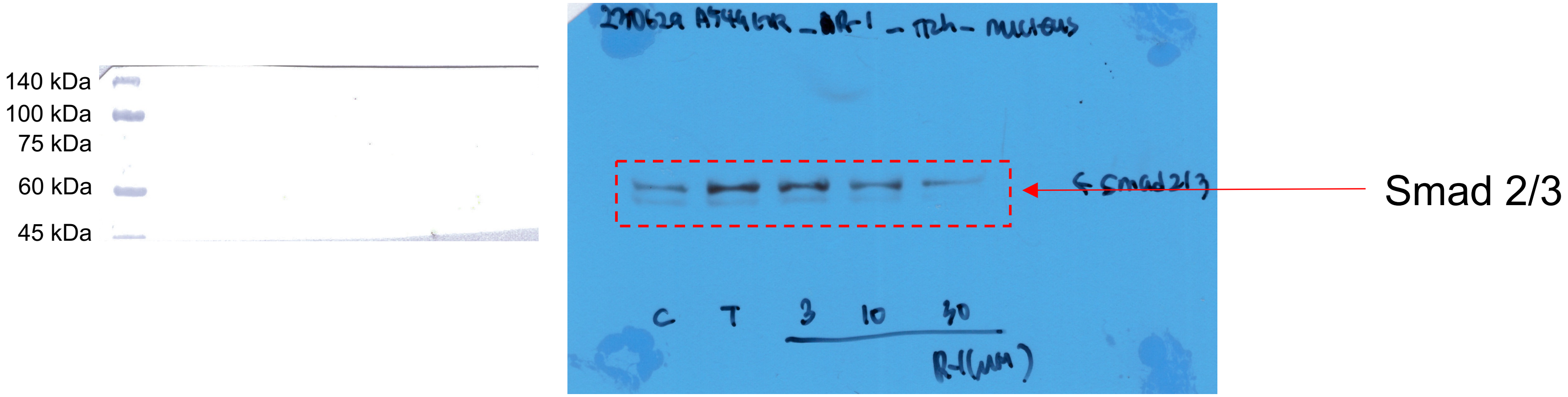

Fig. 2E

A549GR_R-1(cRGDwV)_72 h_nucleus

1. Control 2. TGF-β1 (5ng/ml) 3. TGF-β1 + R-1 3 uM

4. TGF-β1 + R-1 10 uM 5. TGF-β1 + R-1 30 uM

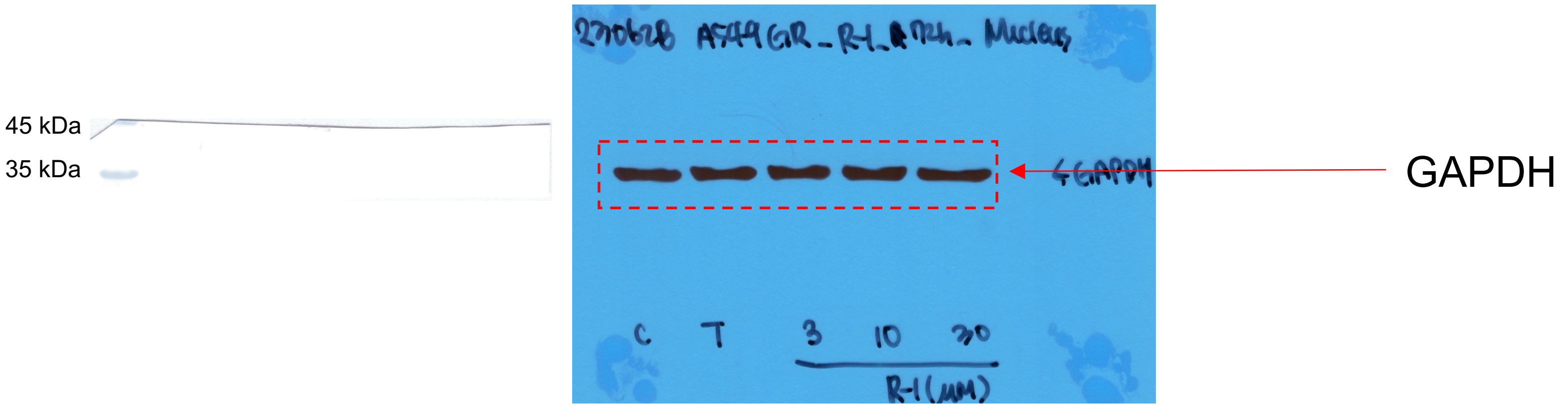

Fig. 2F

A549GR_R-7(cRGDyV)_72 h_nucleus

1. control 2. TGF-β1 (5ng/ml) 3. TGF-β1 + R-7 3uM
4. TGF-β1 + R-7 10uM 5. TGF-β1 + R-7 30uM

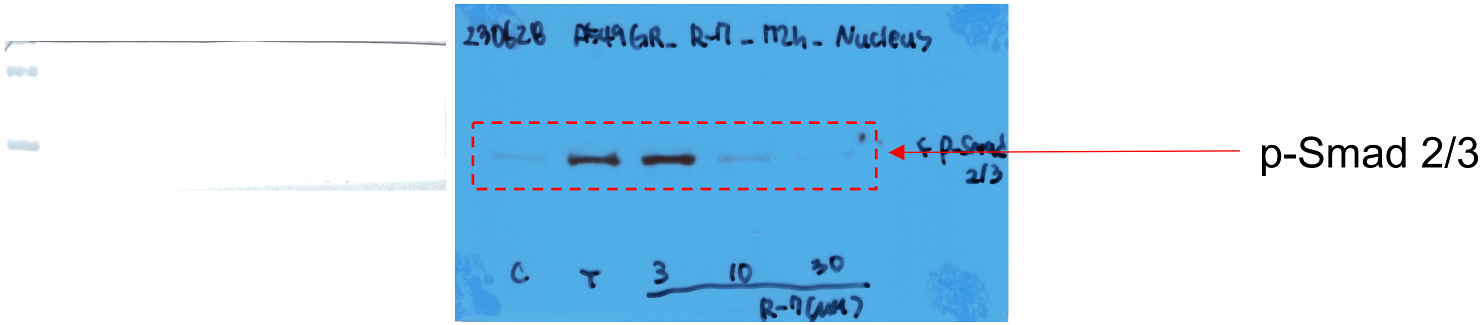

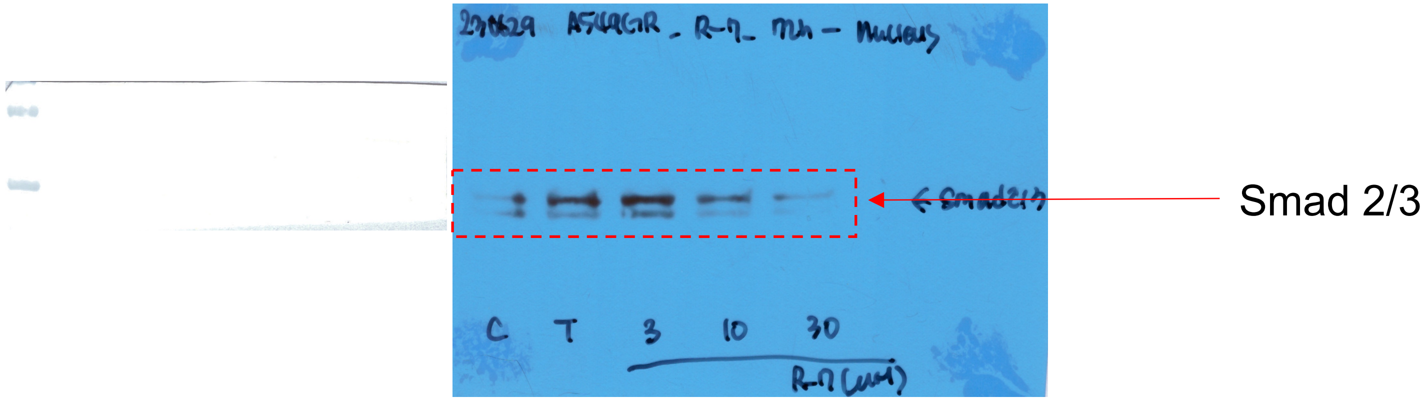

Fig. 2F

A549GR_R-7(cRGDyV)_72 h_nucleus

1. control 2. TGF-β1 (5ng/ml) 3. TGF-β1 + R-7 3uM
4. TGF-β1 + R-7 10uM 5. TGF-β1 + R-7 30uM

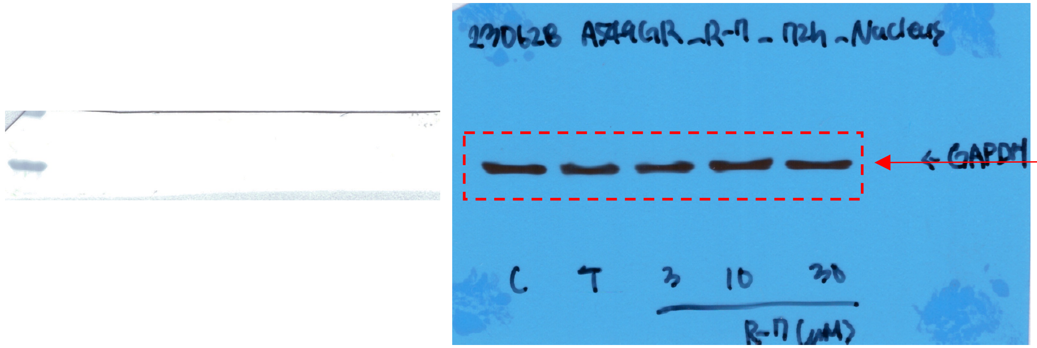

Fig. 4A

A549GR_Gefitinib + R-1(cRGDwV)_72 h_total

1. Control 2. TGF-β1 (5 ng/ml) 3. Gefitinib 1 μM
4. R-1 3 μM 5. Gefitinib 1 μM + R-1 3 μM

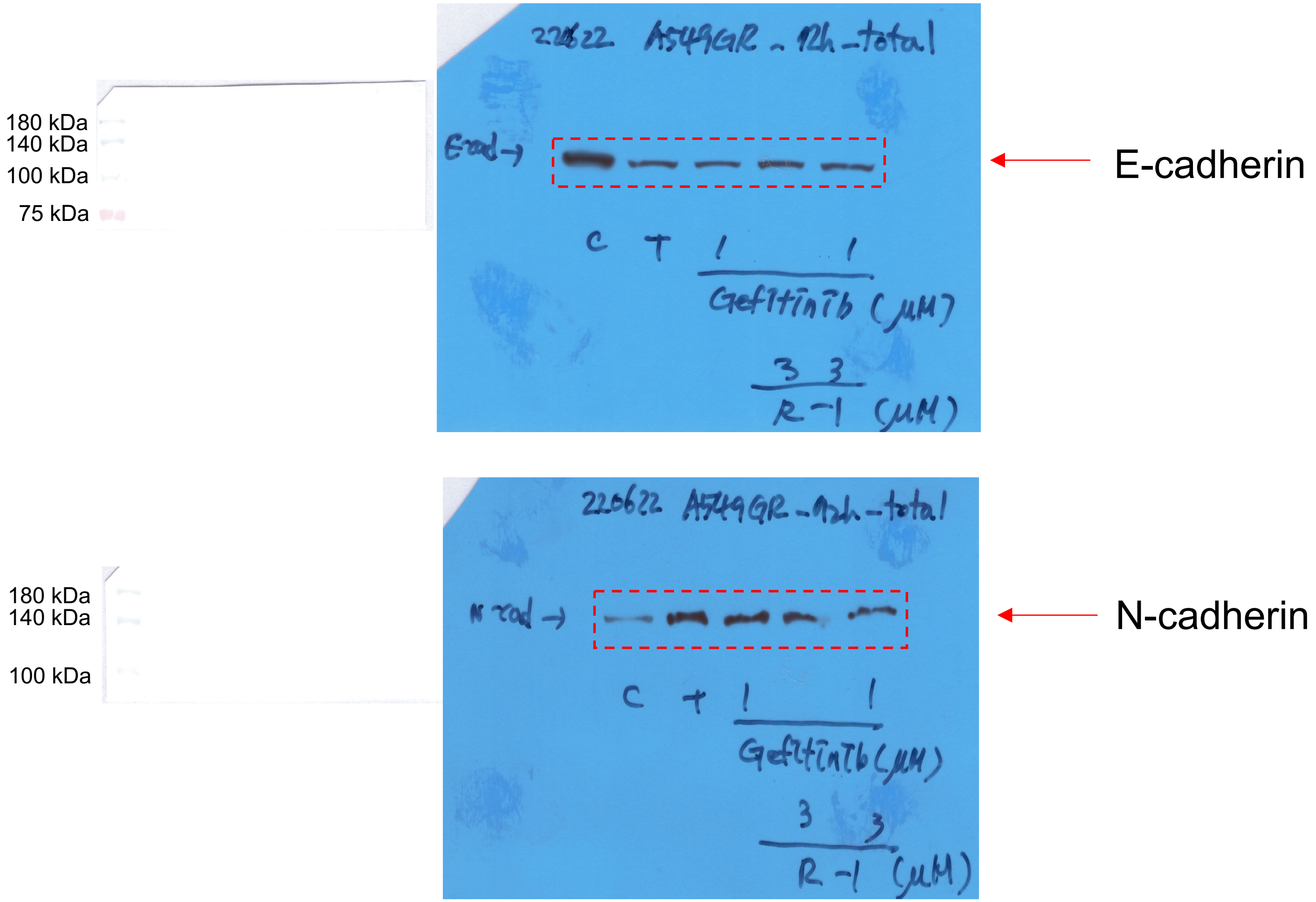

Fig. 4A

A549GR_Gefitinib + R-1(cRGDwV)_72 h_total

1. Control 2. TGF-β1 (5 ng/ml) 3. Gefitinib 1 μM
4. R-1 3 μM 5. Gefitinib 1 μM + R-1 3 μM

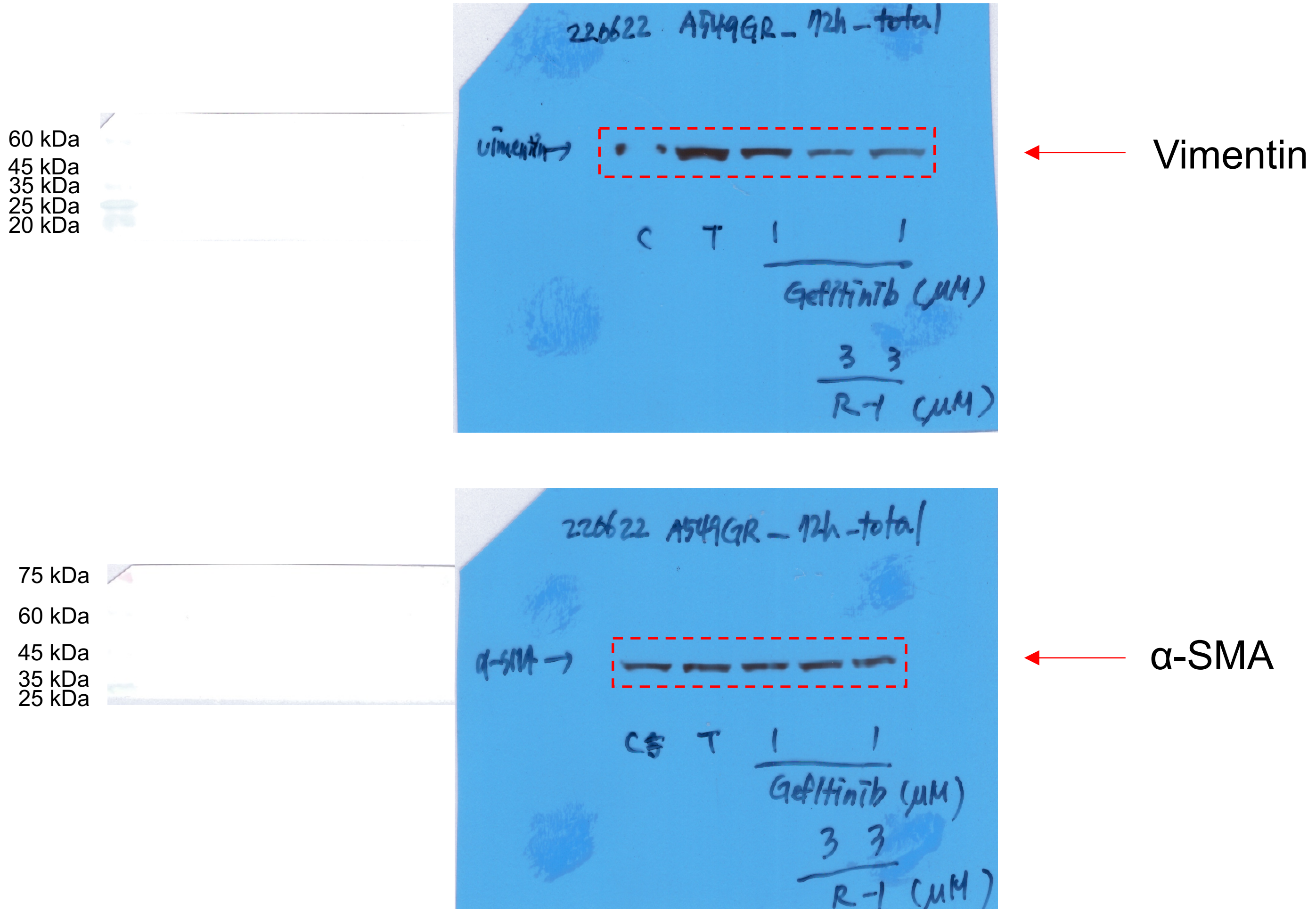

Fig. 4A

A549GR_Gefitinib + R-1(cRGDwV)_72 h_total

1. Control 2. TGF-β1 (5 ng/ml) 3. Gefitinib 1 μM
4. R-1 3 μM 5. Gefitinib 1 μM + R-1 3 μM

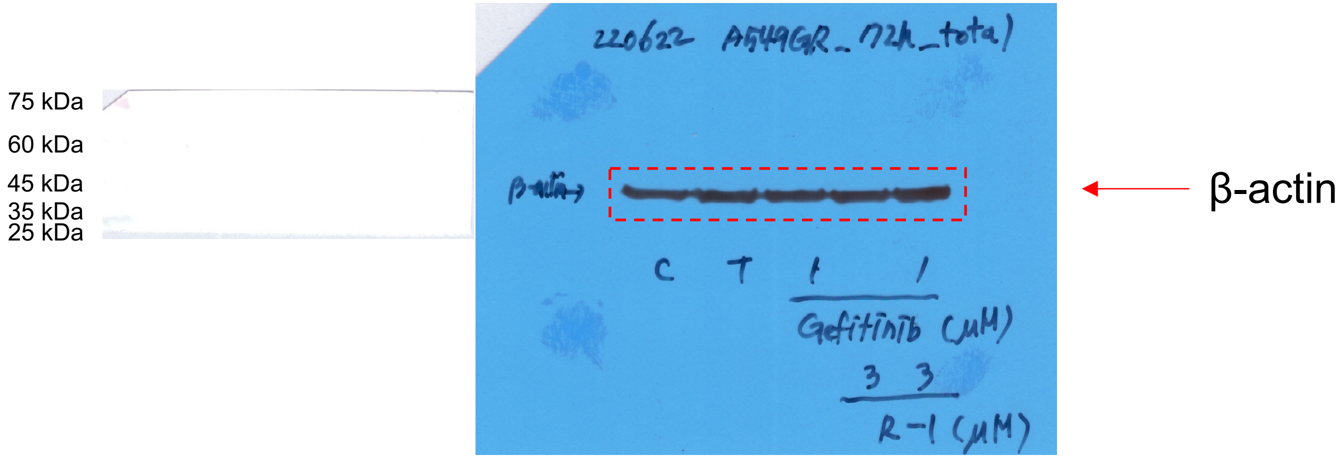

Fig. 4B

A549GR_Gefitinib + R-7(cRGDyV)_72 h_total

1. Control 2. TGF-β1 (5ng/ml) 3. TGF-β1 + Gefitinib 1μM

4. TGF-β1 + R-7 (cRGDyV) 3μM 5. TGF-β1 + Gefitinib 1μM + R-7 3μM

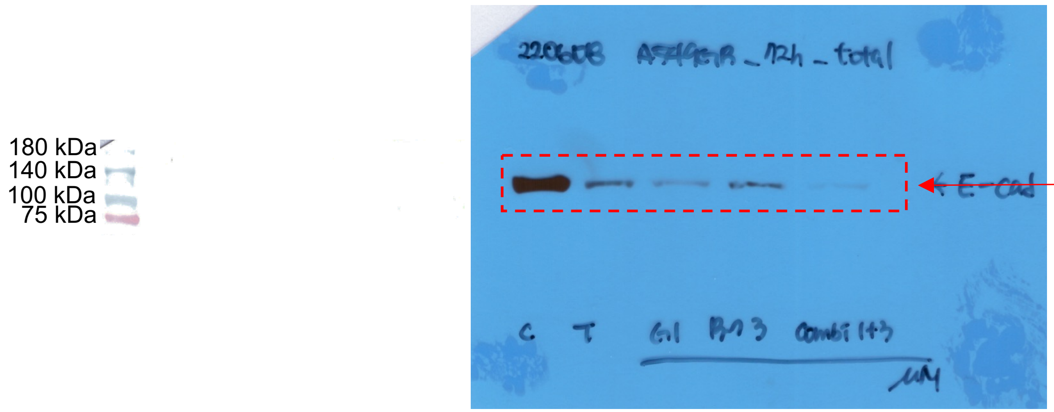

E-cadherin

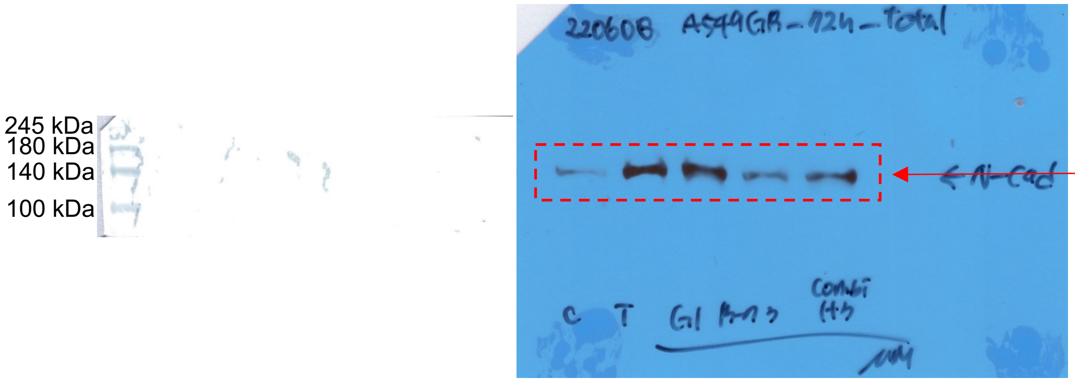

N-cadherin

Fig. 4B

A549GR_Gefitinib + R-7(cRGDyV)_72 h_total

1. Control 2. TGF-β1 (5ng/ml) 3. TGF-β1 + Gefitinib 1μM

4. TGF-β1 + R-7 (cRGDyV) 3μM 5. TGF-β1 + Gefitinib 1μM + R-7 3μM

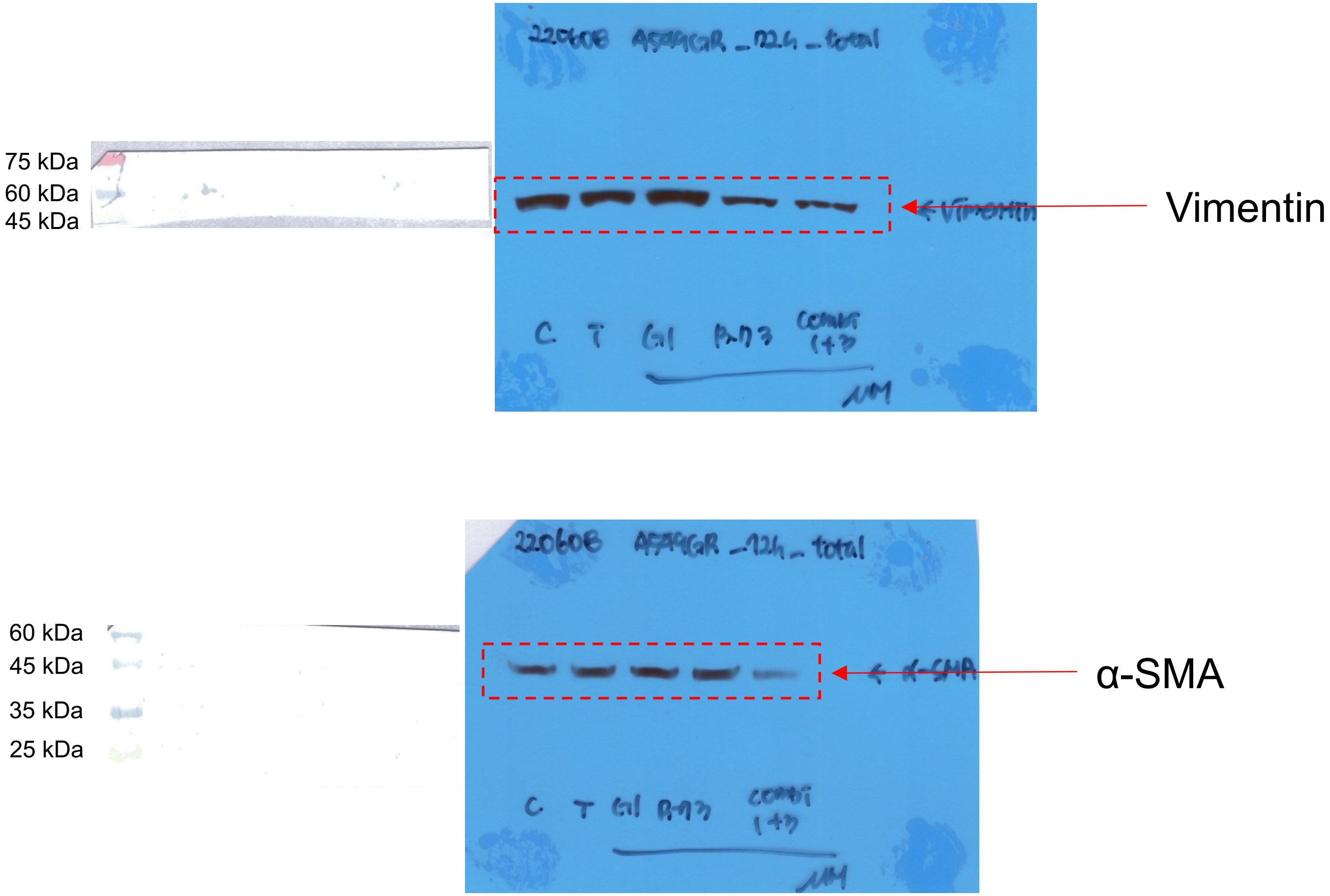

Fig. 4B

A549GR_Gefitinib + R-7(cRGDyV)_72 h_total

1. Control 2. TGF-β1 (5ng/ml) 3. TGF-β1 + Gefitinib 1μM

4. TGF-β1 + R-7 (cRGDyV) 3μM 5. TGF-β1 + Gefitinib 1μM + R-7 3μM

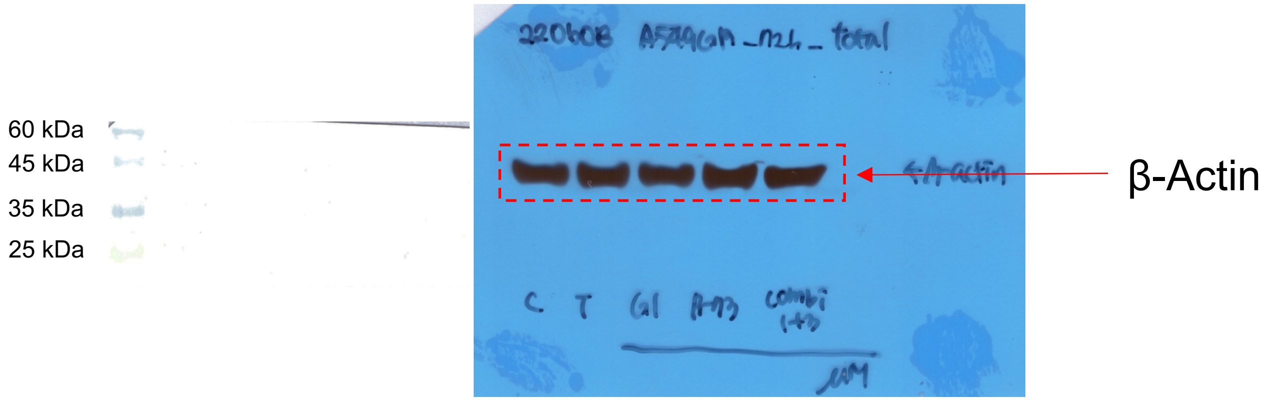

Fig. 4C

A549GR_Gefitinib + R-7(cRGDyV)_72 h_nucleus

1. Control 2. TGF-β1 (5ng/ml) 3. TGF-β1 + Gefitinib 1μM

4. TGF-β1 + R-7 3μM 5. TGF-β1 + Gefitinib 1μM + R-7 3μM

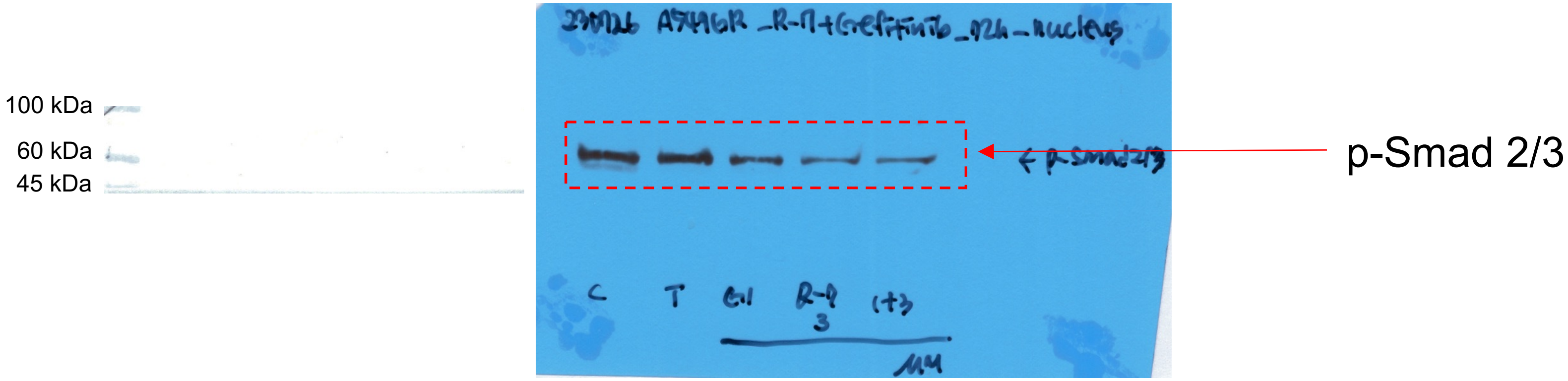

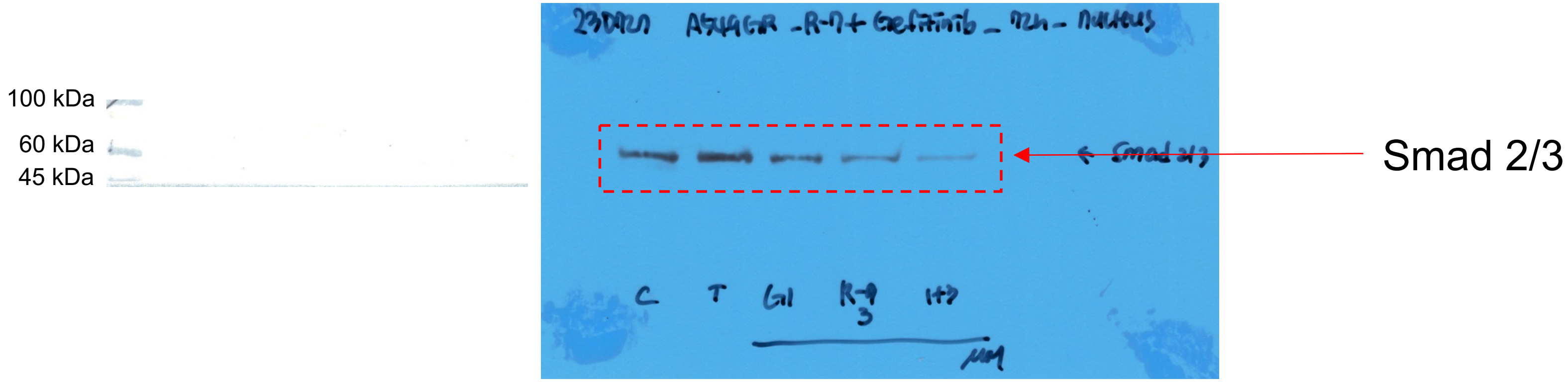

Fig. 4C

A549GR_Gefitinib + R-7(cRGDyV)_72 h_nucleus

1. Control 2. TGF-β1 (5ng/ml) 3. TGF-β1 + Gefitinib 1μM

4. TGF-β1 + R-7 3μM 5. TGF-β1 + Gefitinib 1μM + R-7 3μM

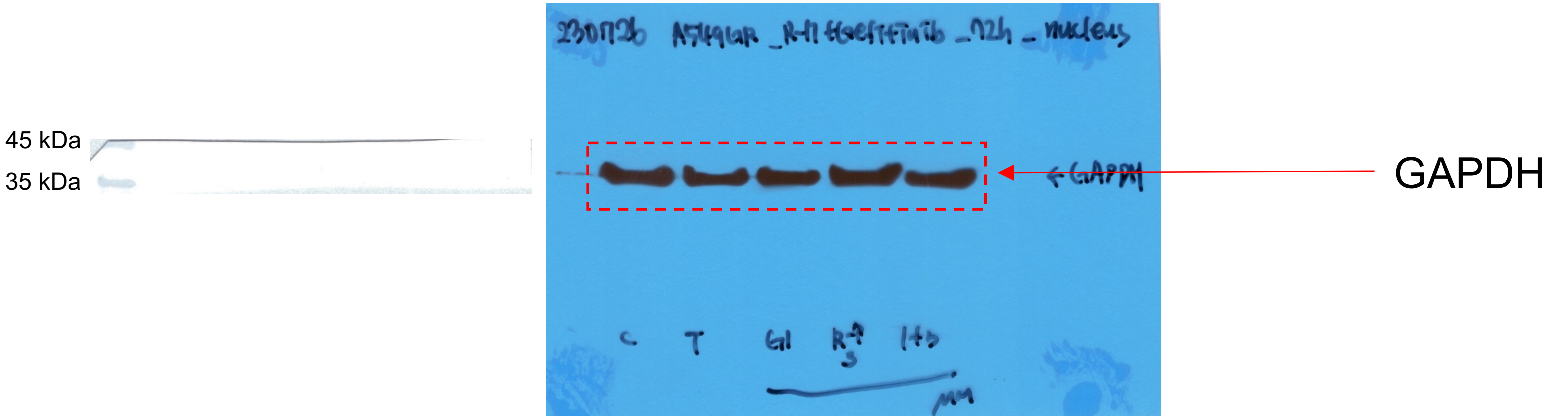

Fig. 4D

A549GR_Gefitinib + R-1(cRGDwV)_72 h_nucleus

1. control 2. TGF-β1 (5ng/ml) 3. Gefitinib 1uM + TGF-β1

4. TGF-β1 + R-1 3uM 5. TGF-β1 + Gefitinib 1uM + R-1 3uM

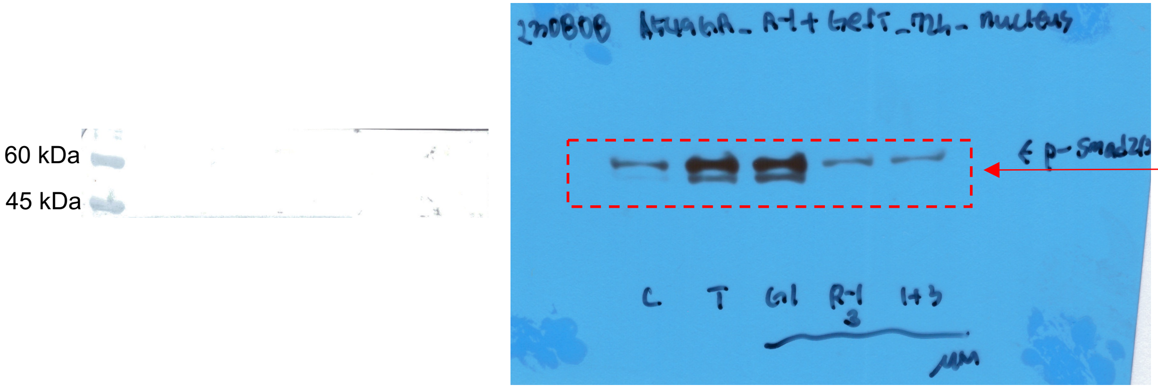

p-Smad 2/3

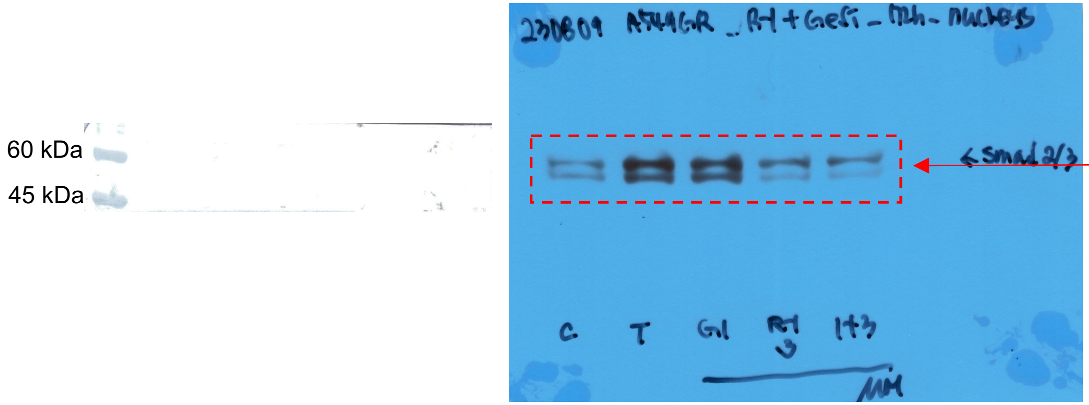

Smad 2/3

Fig. 4D

A549GR_Gefitinib + R-1(cRGDwV)_72 h_nucleus

1. control
2. TGF-β1 (5ng/ml)
3. Gefitinib + 1uM + TGF-β1
4. TGF-β1 + R-1 3uM
5. TGF-β1 + Gefitinib 1uM + R-1 3uM

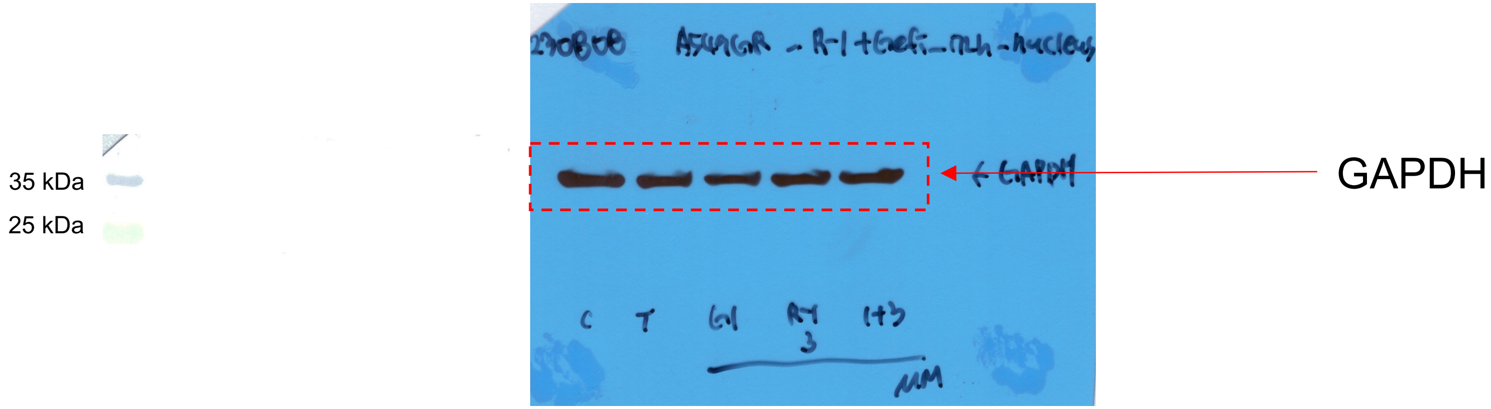

Fig. S20A

A549GR_R-1(cRGDwV)_72 h_ cytosol, nucleus

1. control 2. TGF-β1 (5ng/ml) 3. TGF-β1 + R-1 3 μM

4. TGF-β1 + R-1 10 μM 5. TGF-β1 + R-1 30 μM

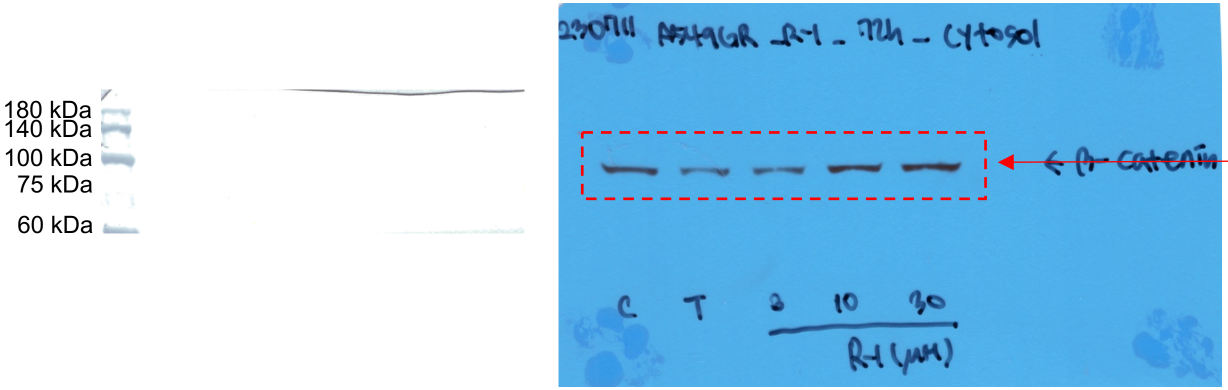

β–catenin(C)

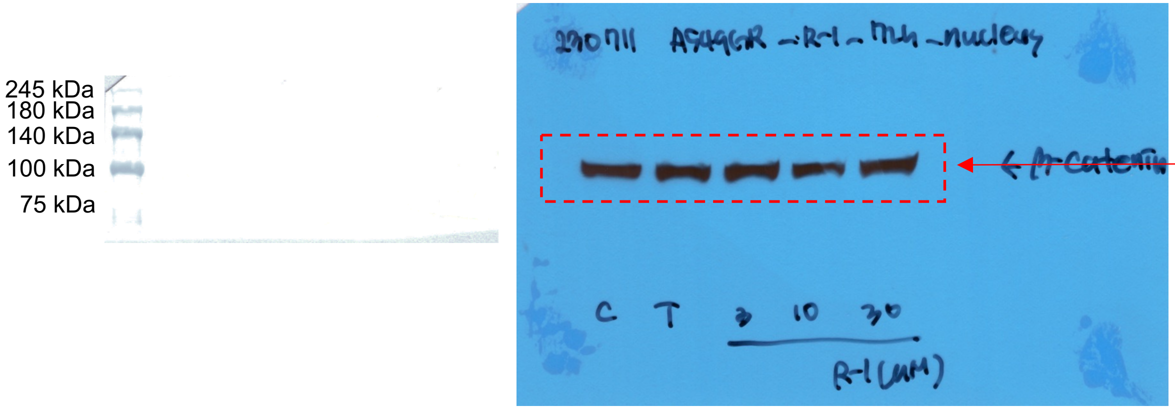

β–catenin(N)

Fig. S20A

A549GR_R-1(cRGDwV)_72 h_ cytosol, nucleus

1. control
2. TGF-β1 (5ng/ml)
3. TGF-β1 + R-1 3 μM
4. TGF-β1 + R-1 10 μM
5. TGF-β1 + R-1 30 μM

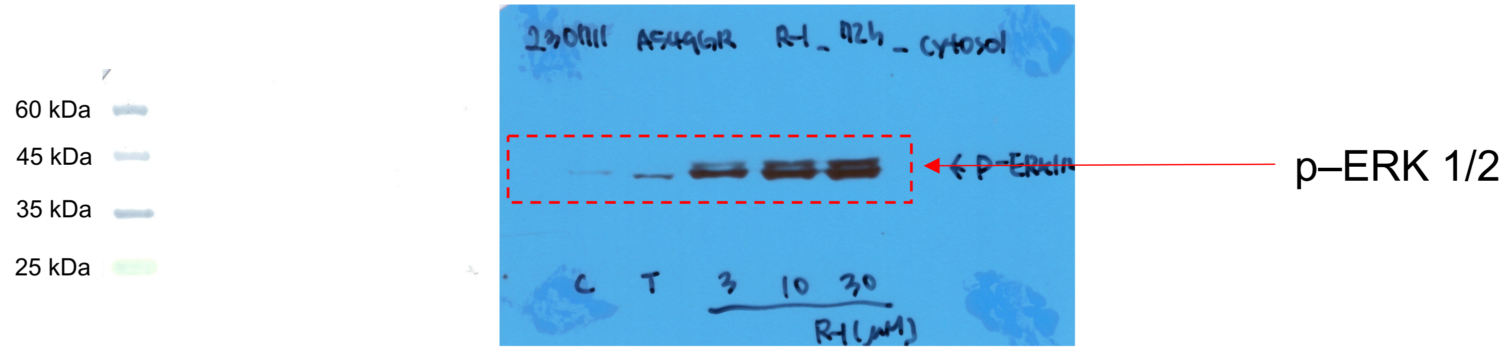

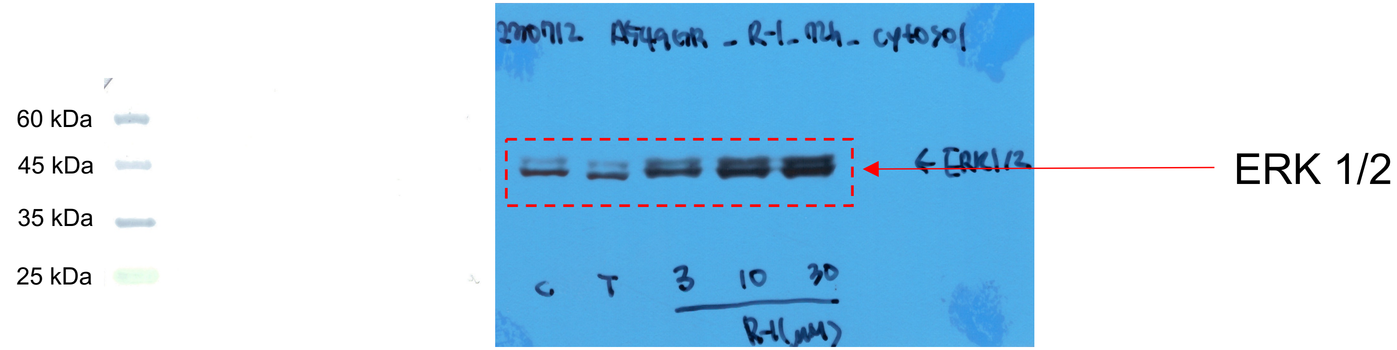

Fig. S20A

A549GR_R-1(cRGDwV)_72 h_ cytosol, nucleus

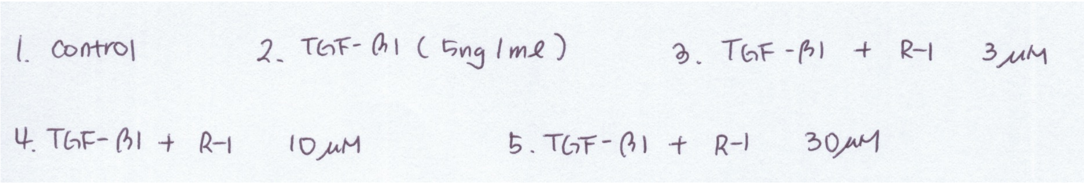

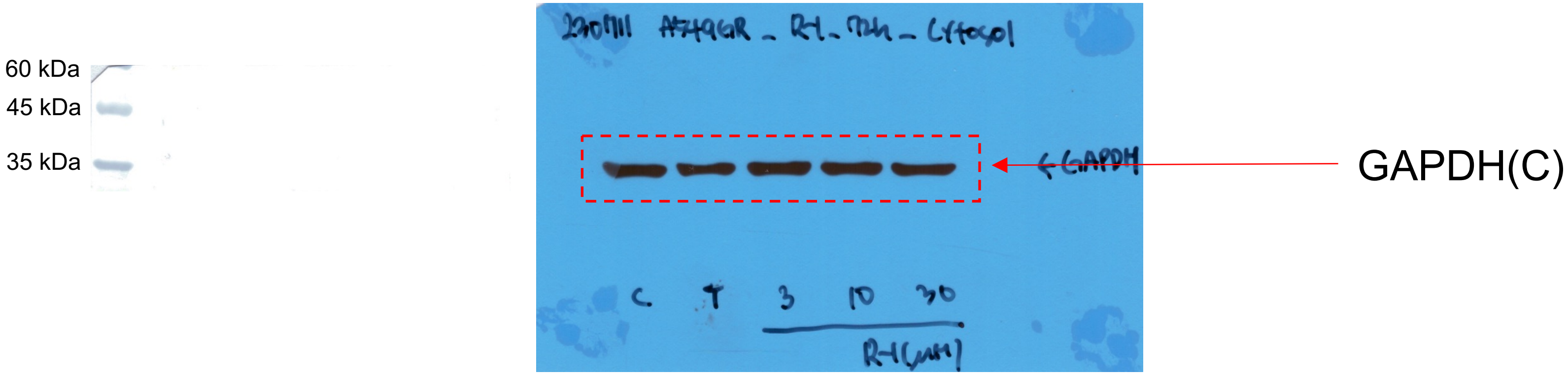

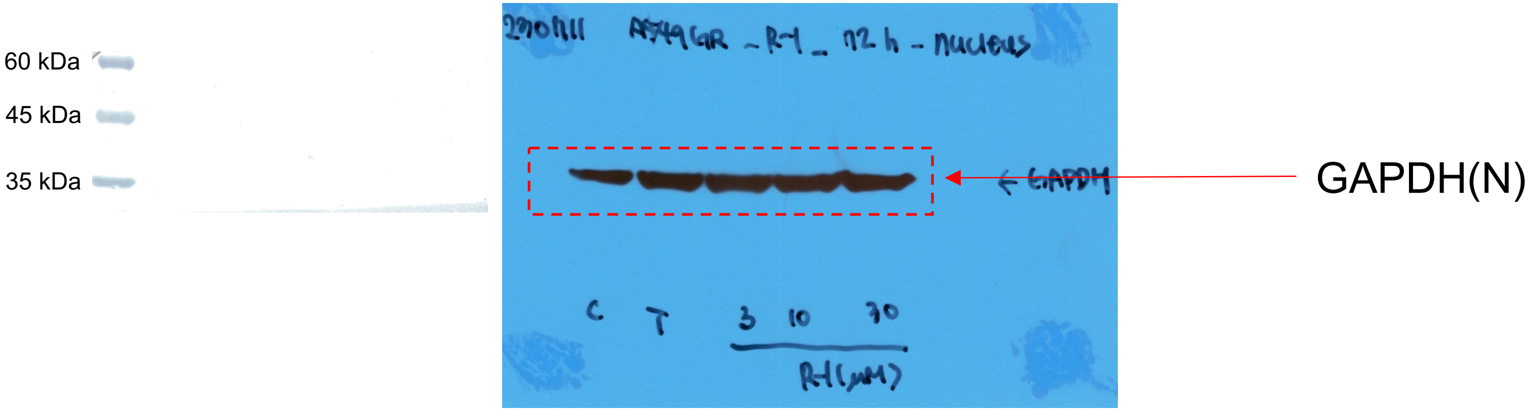

Fig. S20B

A549GR_R-7(cRGDyV)_72 h_ cytosol, nucleus

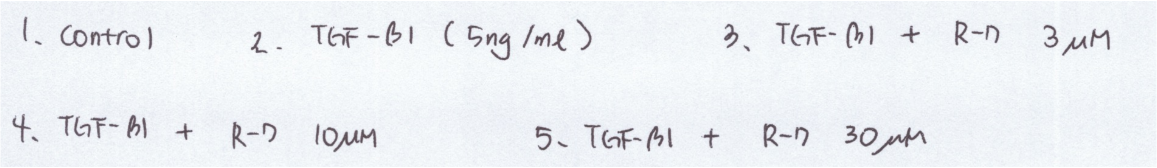

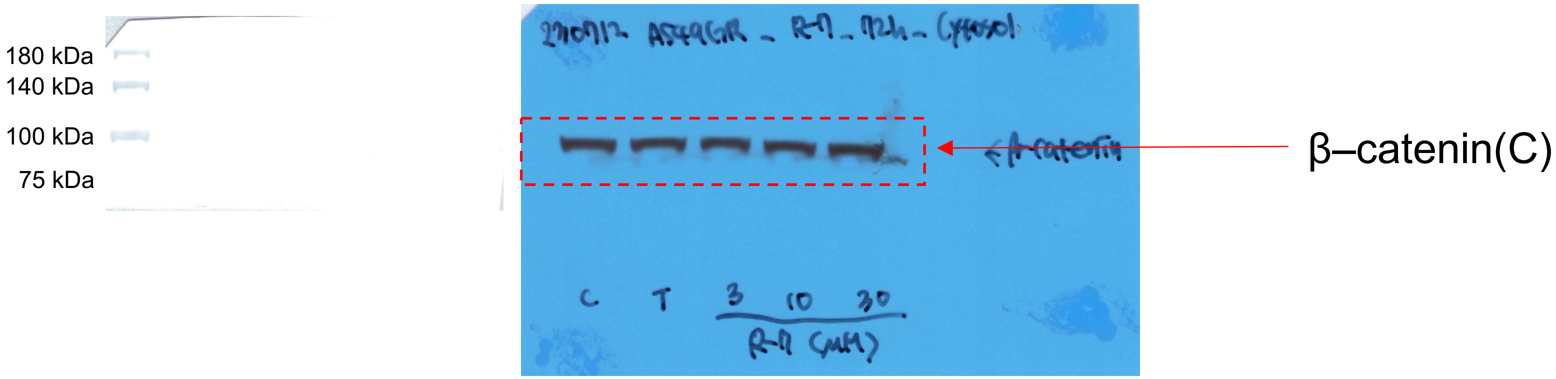

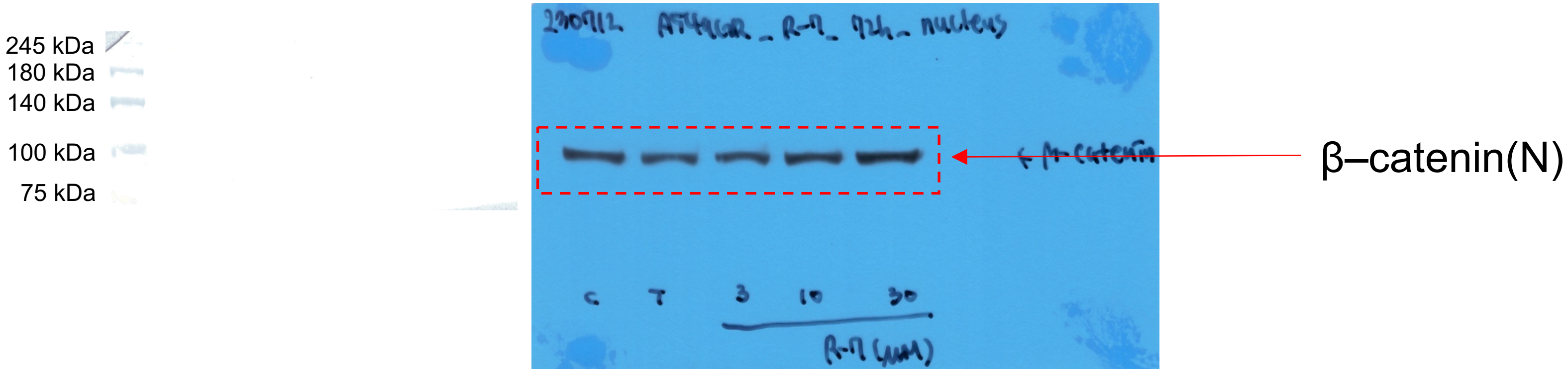

Fig. S20B

A549GR_R-7(cRGDyV)_72 h_ cytosol, nucleus

1. control 2. TGF-β1 (5ng/ml) 3. TGF-β1 + R-7 3 μM

4. TGF-β1 + R-7 10μM 5. TGF-β1 + R-7 30μM

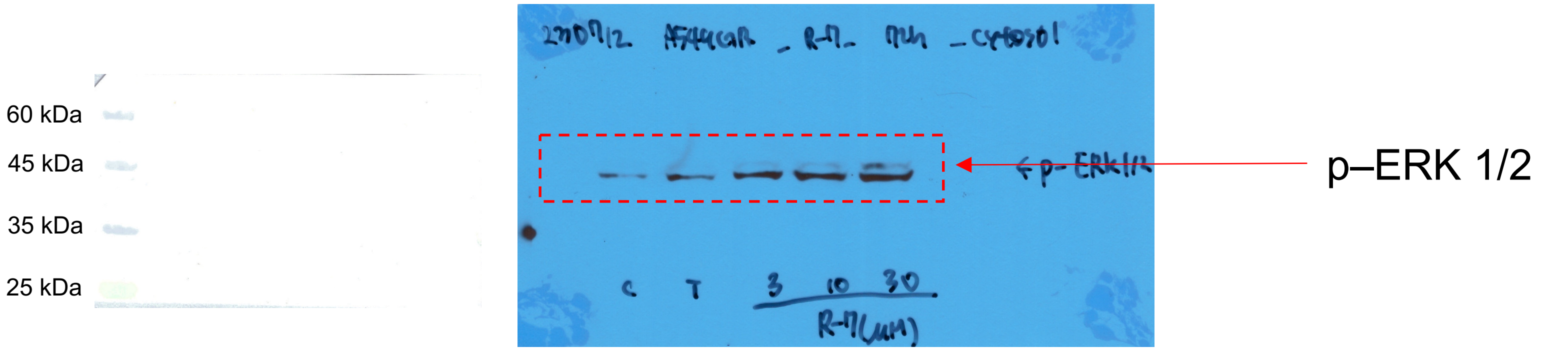

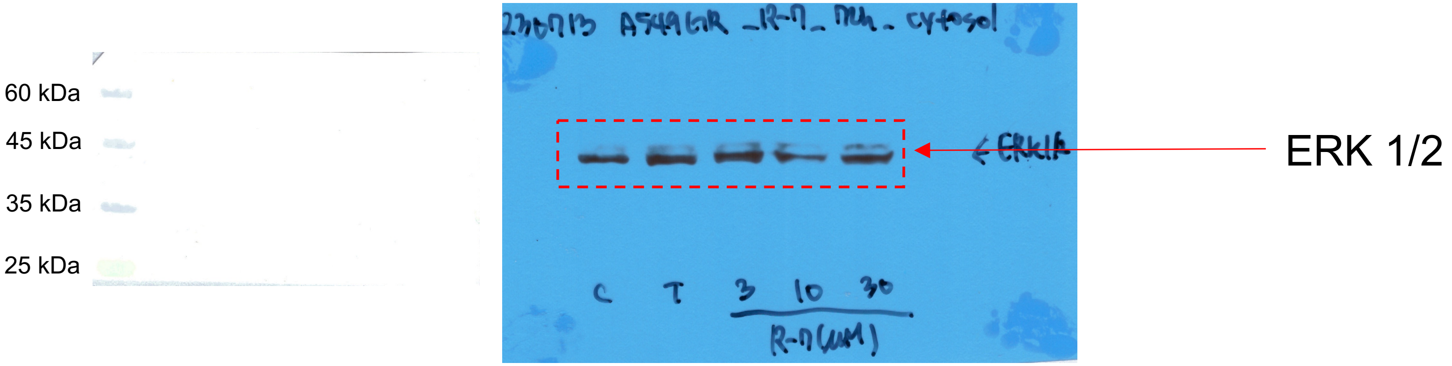

Fig. S20B

A549GR_R-7(cRGDyV)_72 h_ cytosol, nucleus

1. control   2. TGF-β1 (5ng/ml)   3. TGF-β1 + R-7 3 μM

4. TGF-β1 + R-7 10μM   5. TGF-β1 + R-7 30μM

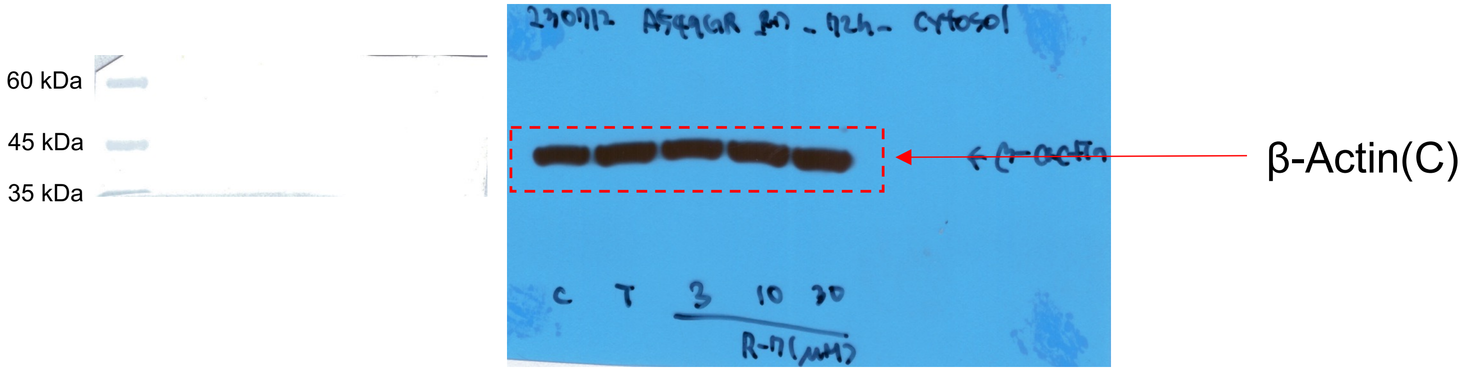

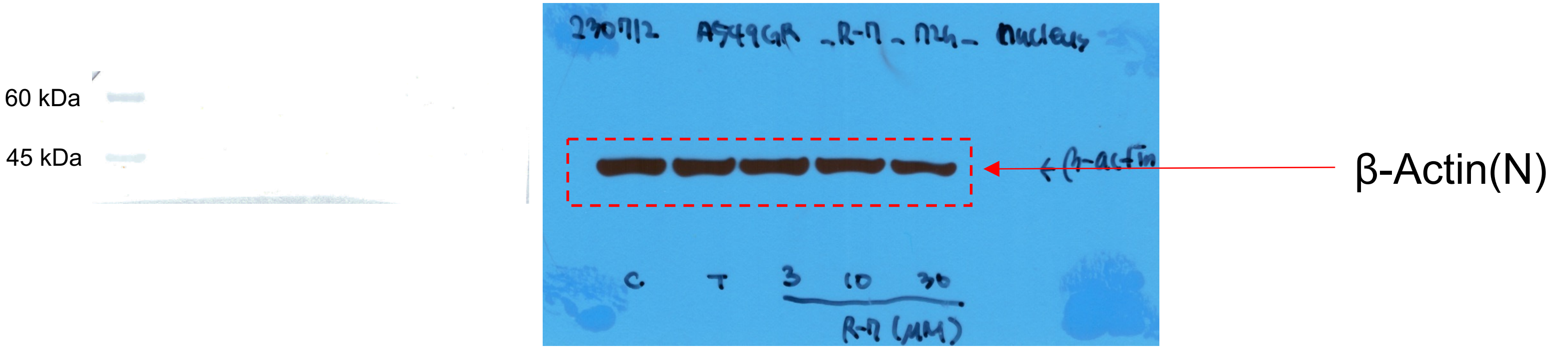

Fig. S21A

A549GR_Gefitinib + R-1(cRGDwV)_72 h_ cytosol

1. control   2. TGF-β1 (5ng/ml)   3. TGF-β1 + Gefitinib 1μM

4. TGF-β1 + R-1 3μM   5. TGF-β1 + Gefitinib 1μM + R-1 3μM

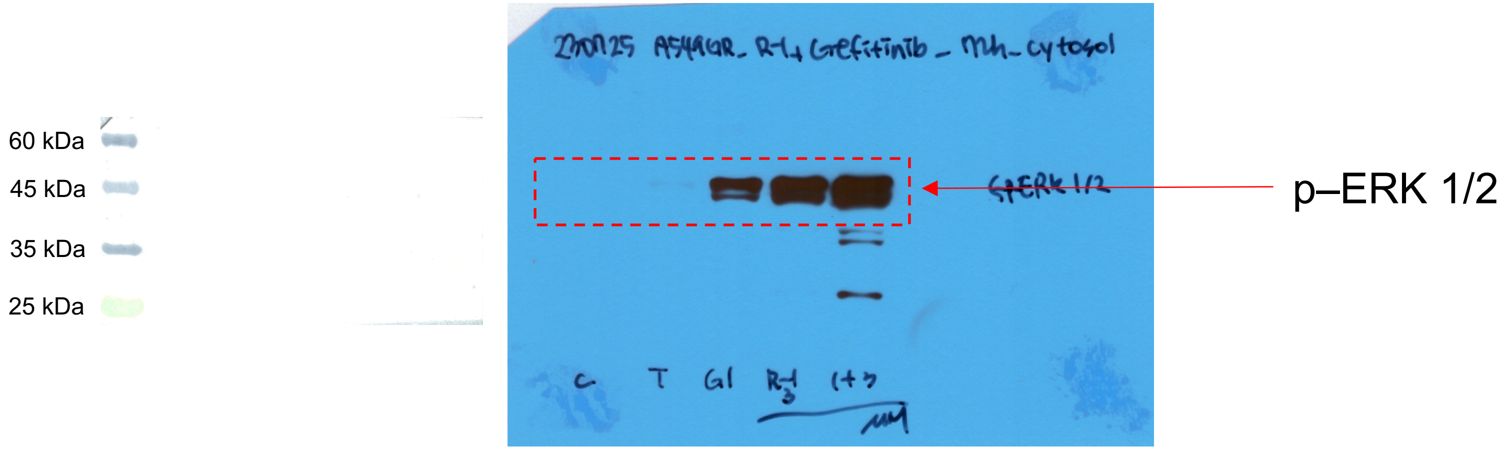

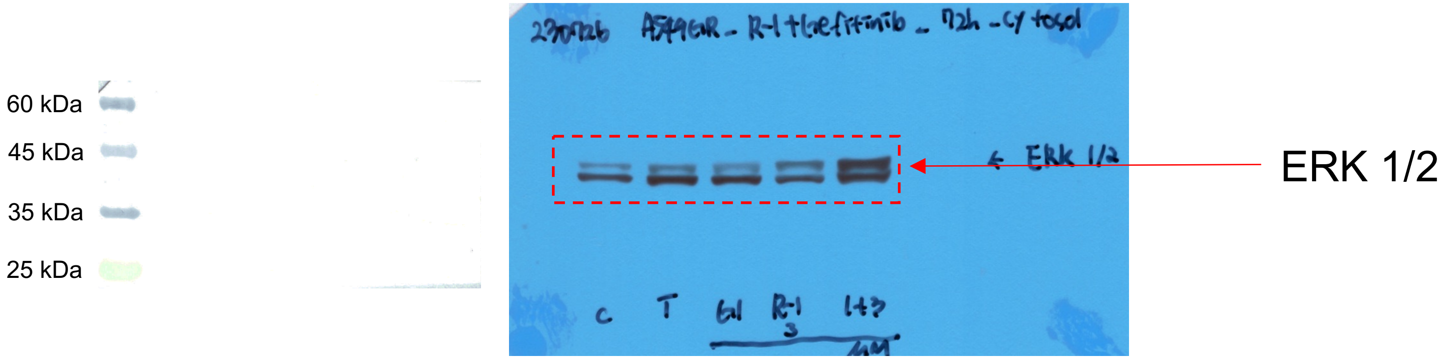

Fig. S21A

A549GR_Gefitinib + R-1(cRGDwV)_72 h_ cytosol

1. control 2. TGF-β1 (5ng/mℓ) 3. TGF-β1 + Gefitinib 1μM

4. TGF-β1 + R-1 3μM 5. TGF-β1 + Gefitinib 1μM + R-1 3μM

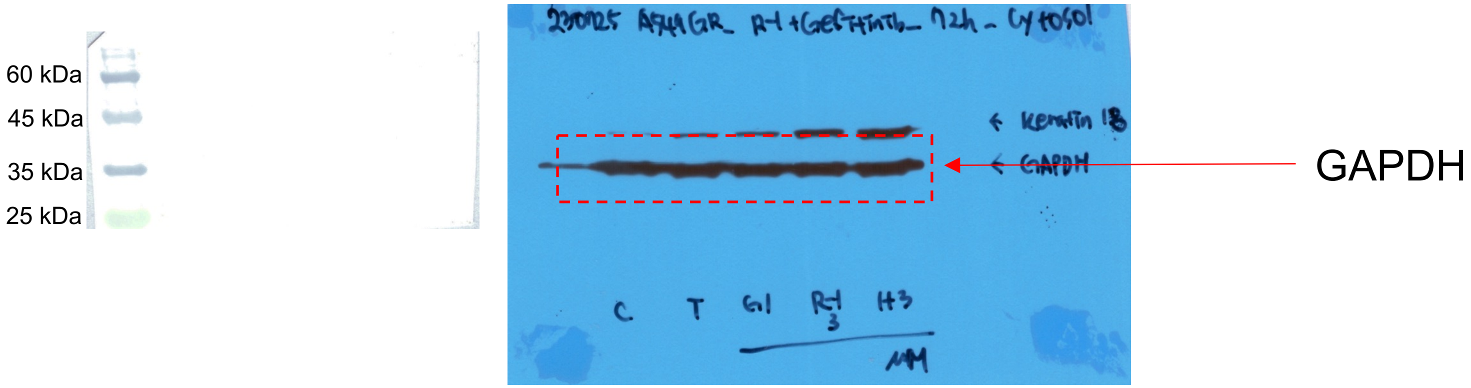

Fig. S21B

A549GR_Gefitinib + R-1(cRGDwV)_72 h_ cytosol, nucleus

1. control 2. TGF-β1 (5ng/ml) 3. TGF-β1 + Gefitinib 1uM

4. TGF-β1 + R-1 (cRGDwV) 3uM 5. TGF-β1 + Gefitinib 1uM + R-1 3uM

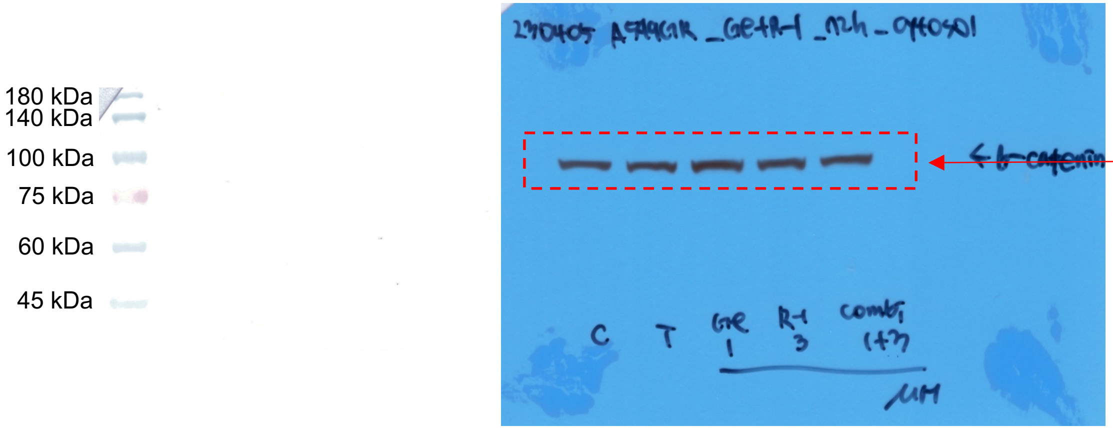

β-catenin(C)

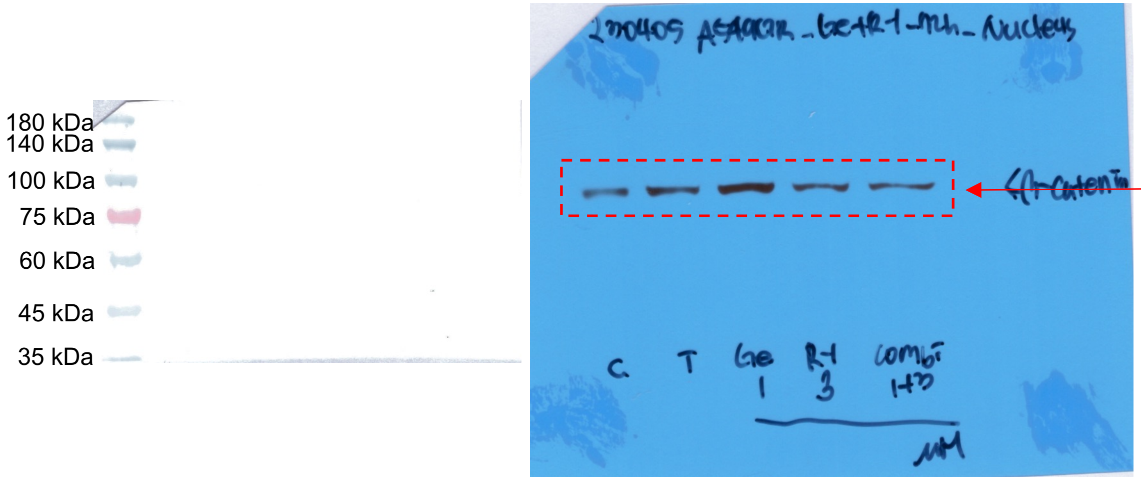

β-catenin(N)

Fig. S21B

A549GR_Gefitinib + R-1(cRGDwV)_72 h_ cytosol, nucleus

1. control 2. TGF-β1 (5ng/ml) 3. TGF-β1 + Gefitinib 1uM

4. TGF-β1 + R-1 (cRGDwV) 3uM 5. TGF-β1 + Gefitinib 1uM + R-1 3uM

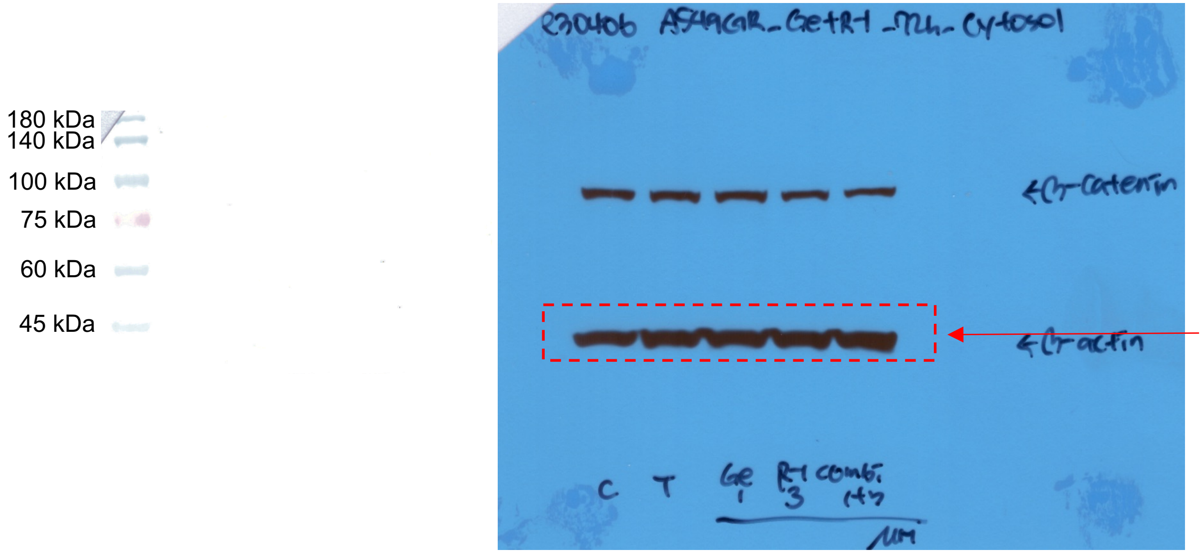

β-actin(C)

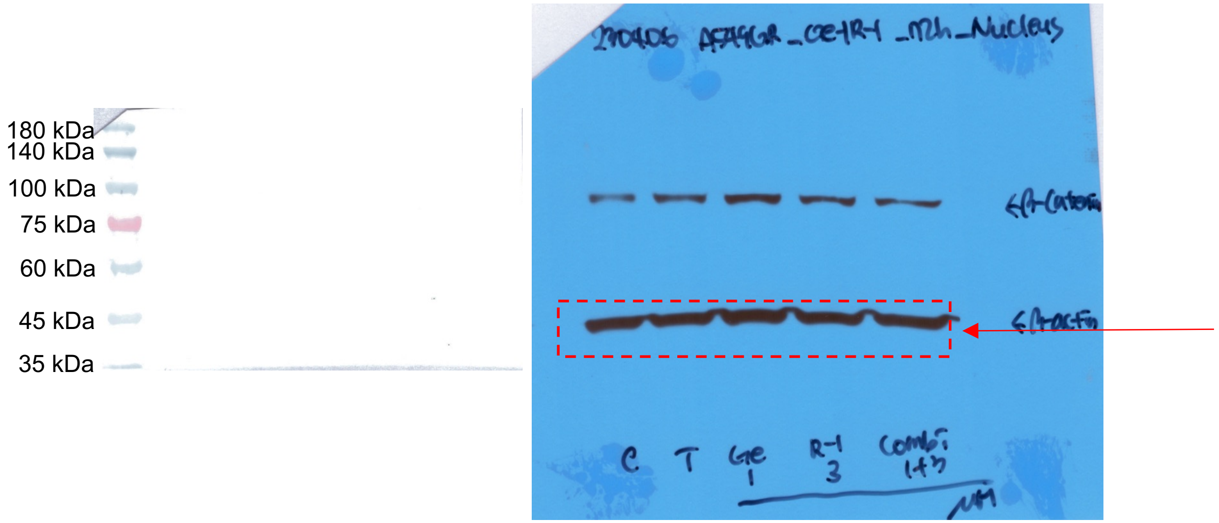

β-actin(N)

A549GR_Gefitinib + R-7(cRGDyV)_72 h_ cytosol

1. control 2. TGF-β1 (5ng/ml) 3. TGF-β1 + Gefitinib 1uM

4. TGF-β1 + R-7 3uM 5. TGF-β1 + Gefitinib 1uM + R-7 3uM

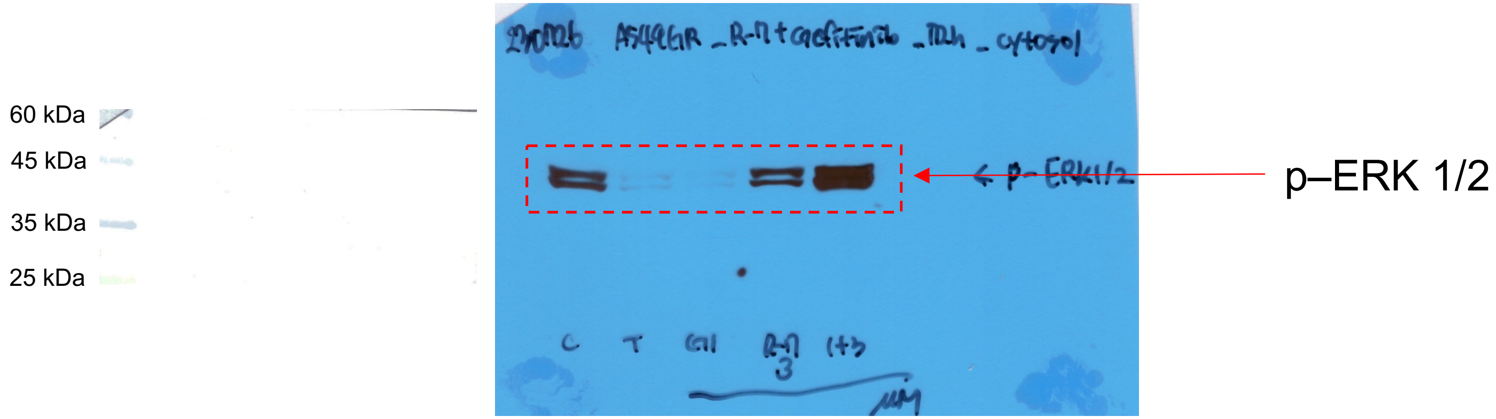

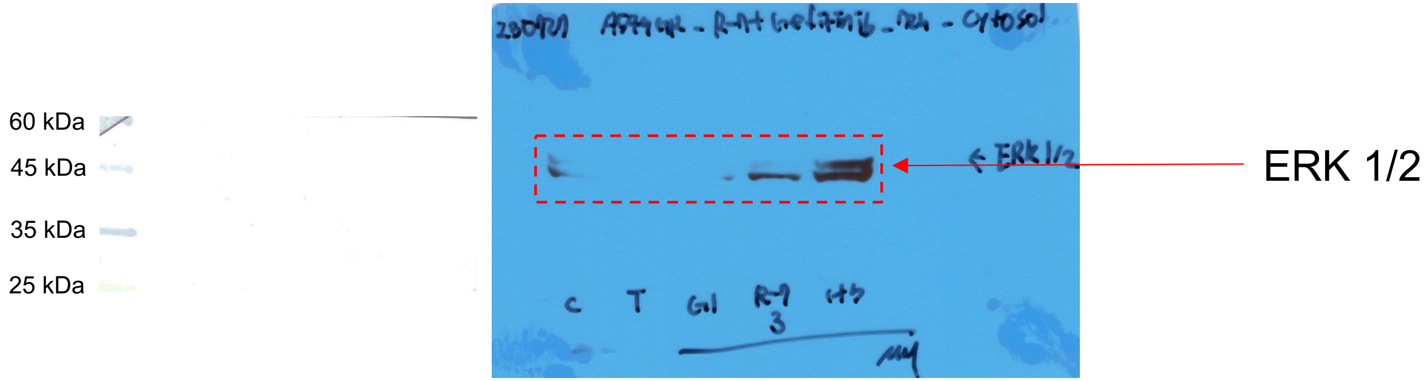

Fig. S21C

A549GR_Gefitinib + R-7(cRGDyV)_72 h_ cytosol

1. control 2. TGF-β1 (5ng/ml) 3. TGF-β1 + Gefitinib 1uM

4. TGF-β1 + R-7 3uM 5. TGF-β1 + Gefitinib 1uM + R-7 3uM

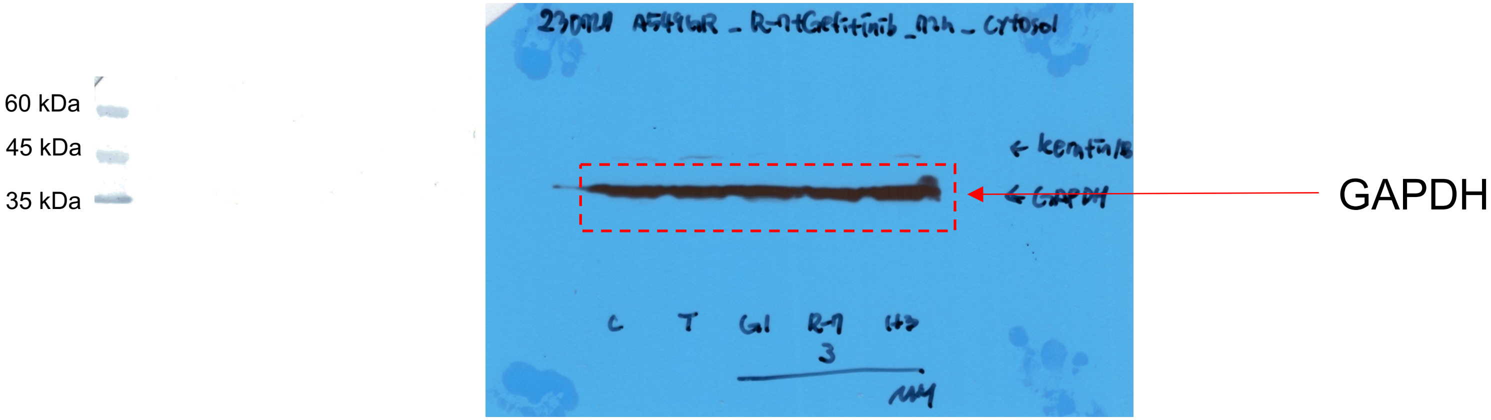

Fig. S21D

A549GR_Gefitinib + R-7(cRGDyV)_72 h_ cytosol, nucleus

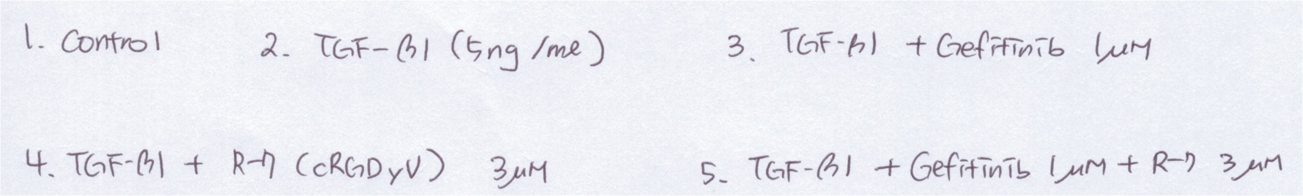

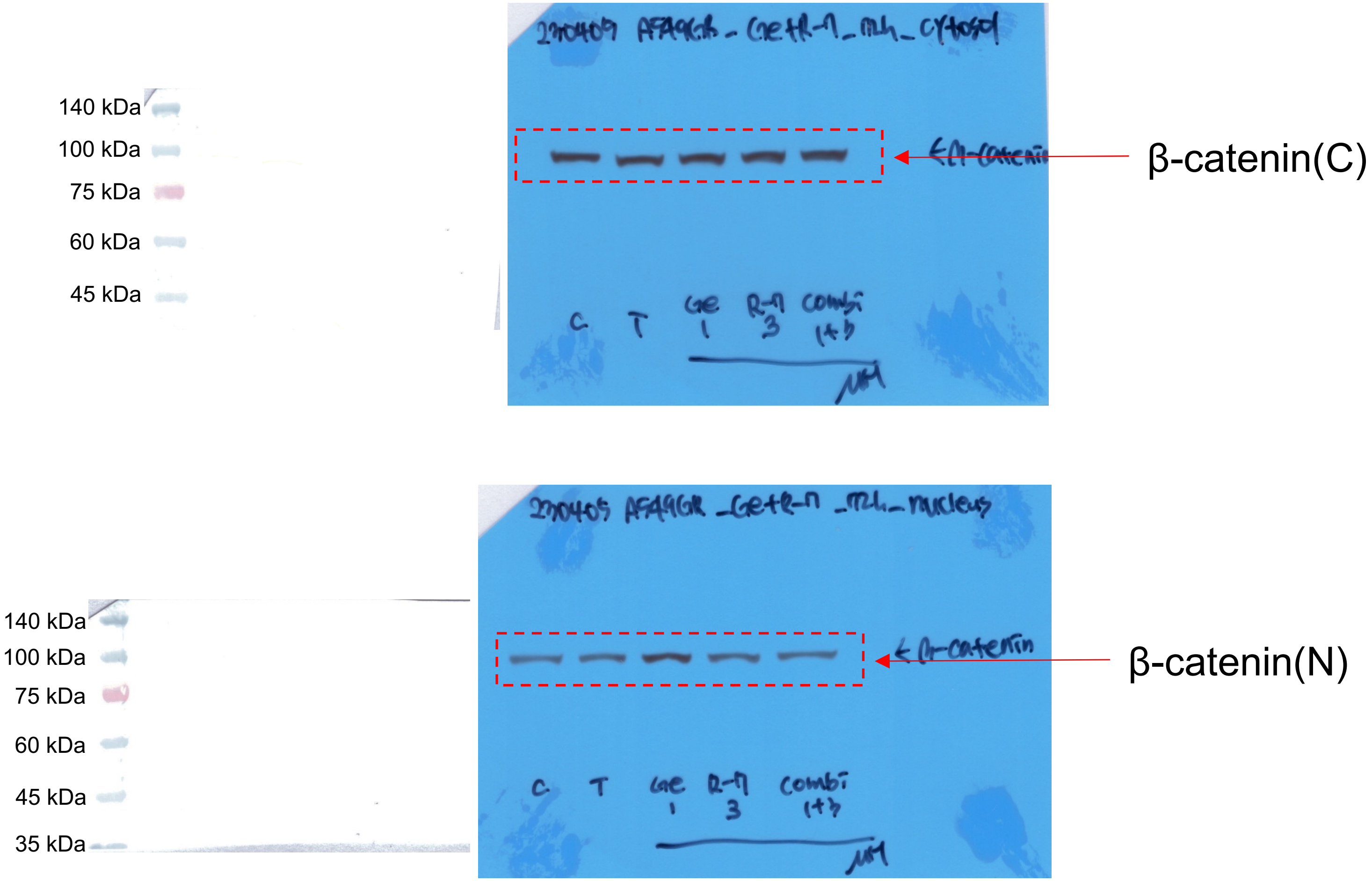

Fig. S21D

A549GR_Gefitinib + R-7(cRGDyV)_72 h_ cytosol, nucleus

1. Control 2. TGF-β1 (5ng/ml) 3. TGF-β1 + Gefitinib 1uM

4. TGF-β1 + R-7 (cRGDyV) 3uM 5. TGF-β1 + Gefitinib 1uM + R-7 3uM

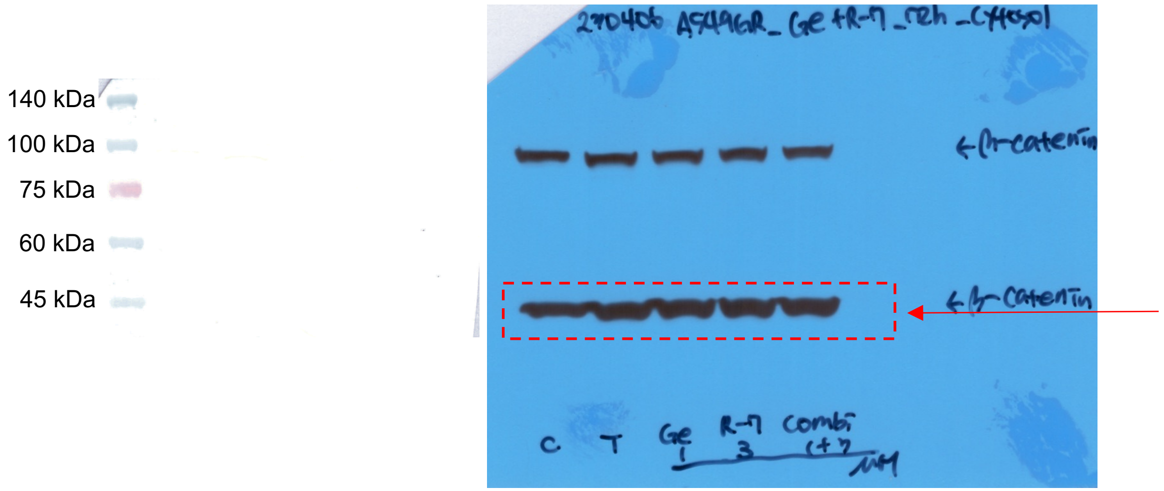

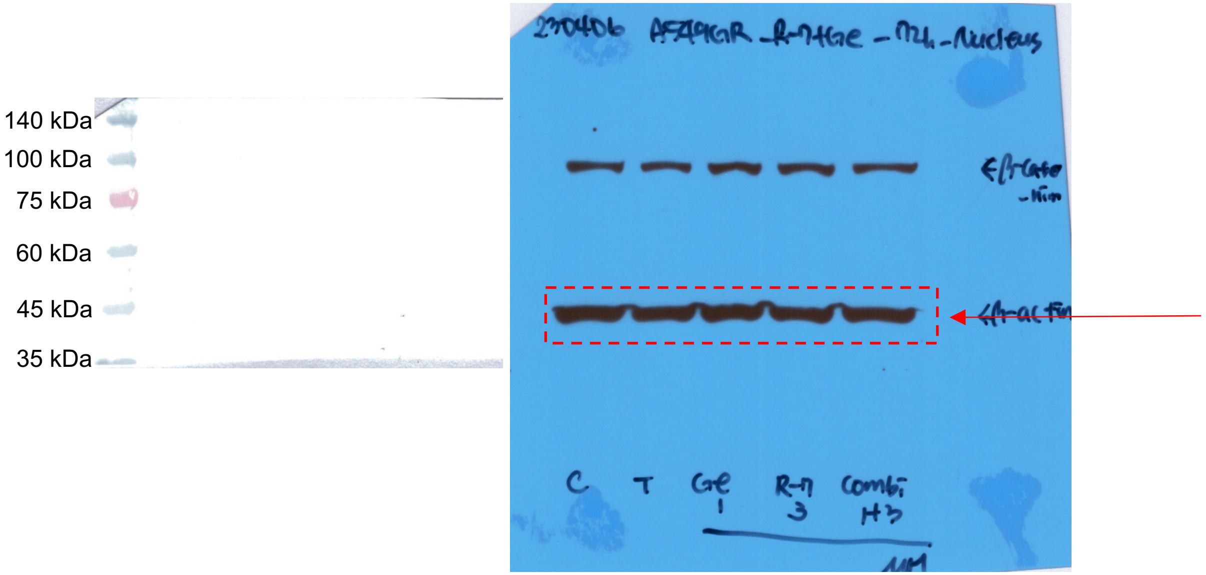

Supplement: Supplementary file 1 [file DataSheet2.pdf]
