# Supplementary material for: Effects of cilengitide derivatives on TGF-β1-induced epithelial-to-mesenchymal transition and invasion in gefitinib-resistant non-small cell lung cancer cells
Source: Front Pharmacol. 2023 Oct 20;14:1277199. doi: 10.3389/fphar.2023.1277199 (PMC10622769; doi:10.3389/fphar.2023.1277199)
Supplement: Supplementary file 2 [file DataSheet1.docx]

**Title:** Effects of cilengitide derivatives on TGF-β1-induced epithelial-to-mesenchymal transition and invasion in gefitinib-resistant non-small cell lung cancer cells

**Experimental Section**

**Mass spectrometry**

Cilengitide derivative masses were determined on a Waters Acquity UPLC H-Class/SQD2

**HPLC spectrometry**

HPLC analysis of cilengitide derivative peptides was conducted in an H_2_O/acetonitrile 1:1 (v/v) solution (1 mg/mL) using a Shimadzu HPLC 2030 model. Analysis conditions were as follows:

| - Instrument | : Shimadzu HPLC 2030 system |
| --- | --- |
| - Flow Rate | : 1 mL/min |
| - Gradient | : 0%–0.3% B in 30 min; 30%–60% B in 33 min; 60% B in 35 min |
| - Buffer | : A buffer: 0.1% TFA in H_2_O; B buffer: 0.1% TFA in CH_3_CN |
| - Column | : Vydac 218TP C18, 5 um, 4.6 × 250 mm |


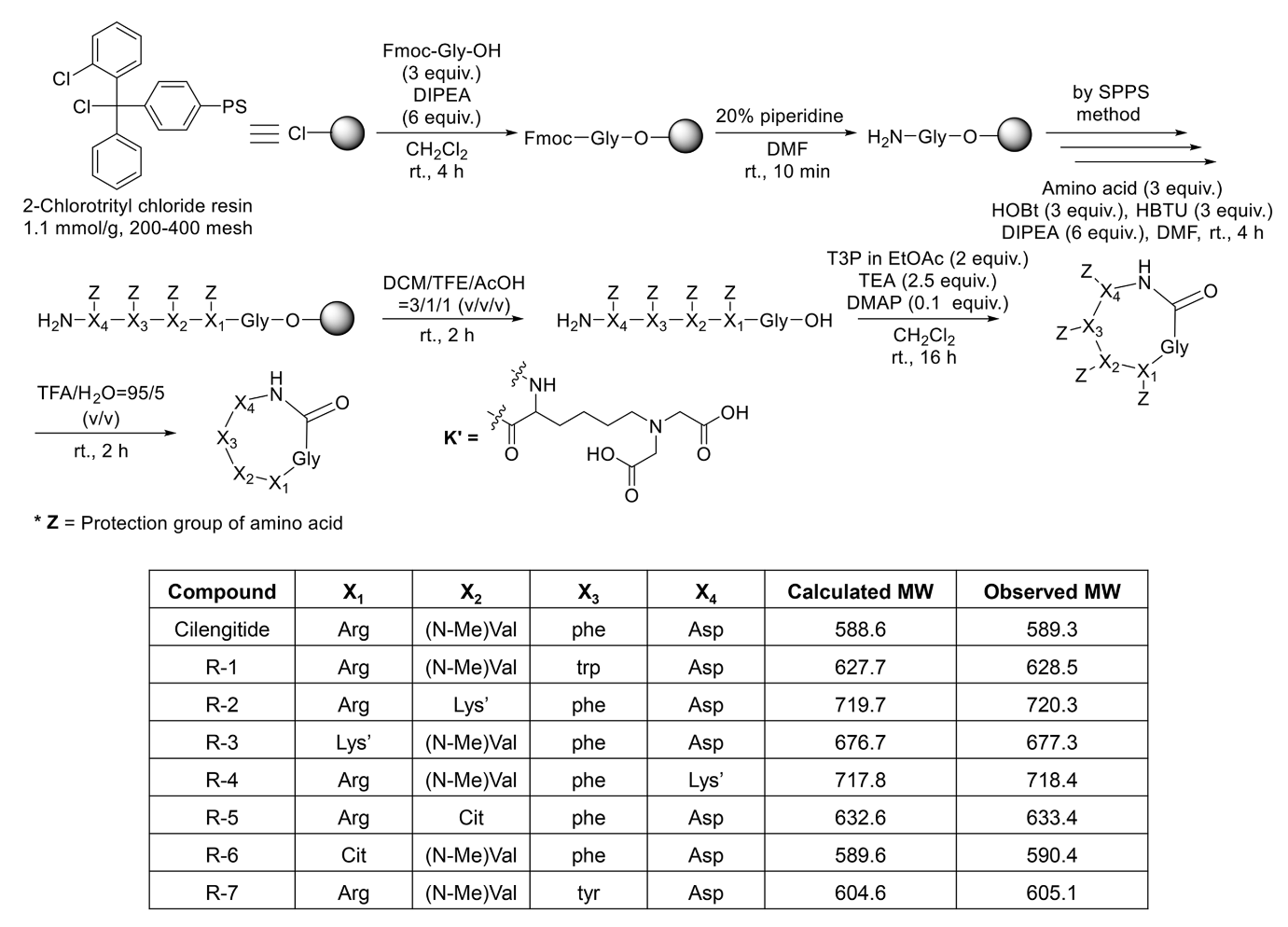


**Figure S1.** Solid phase synthesis of c[RGDf(NMe)V] (cilengitide) derivatives. The protected linear pentapeptides (**1**) bound to the resin were synthesized by an Fmoc solid-phase peptide synthesis method using 2-chlorotrityl chloride resin (loading capacity: 1.01 mmol). At this time, the amino acids of X_3_ were used in the D-form, and the lysine derivatives (K′ = Nα-Fmoc-Nε,Nε-bis(*tert*-butyloxycarbonyl-methyl)-L-lysine) were synthesized by use of a published procedure. The linear peptide (**2**) was cleaved from the resin surface under an acetic acid/TFE/CH_2_Cl_2_ (1:1:3 ratio) solution. Finally, cilengitide derivatives (**4**) were obtained by head-to-tail cyclization under T3P, TEA, DAMP, and elimination of the protecting group using a TFA/H_2_O 95:5 (v/v) solution. The crude peptides were purified by preparative HPLC (Shimadzu LC-20A, Japan) and the molecular weights of the cilengitide derivatives were confirmed using LC-Mass Spectrometry (Agilent 6460, USA).


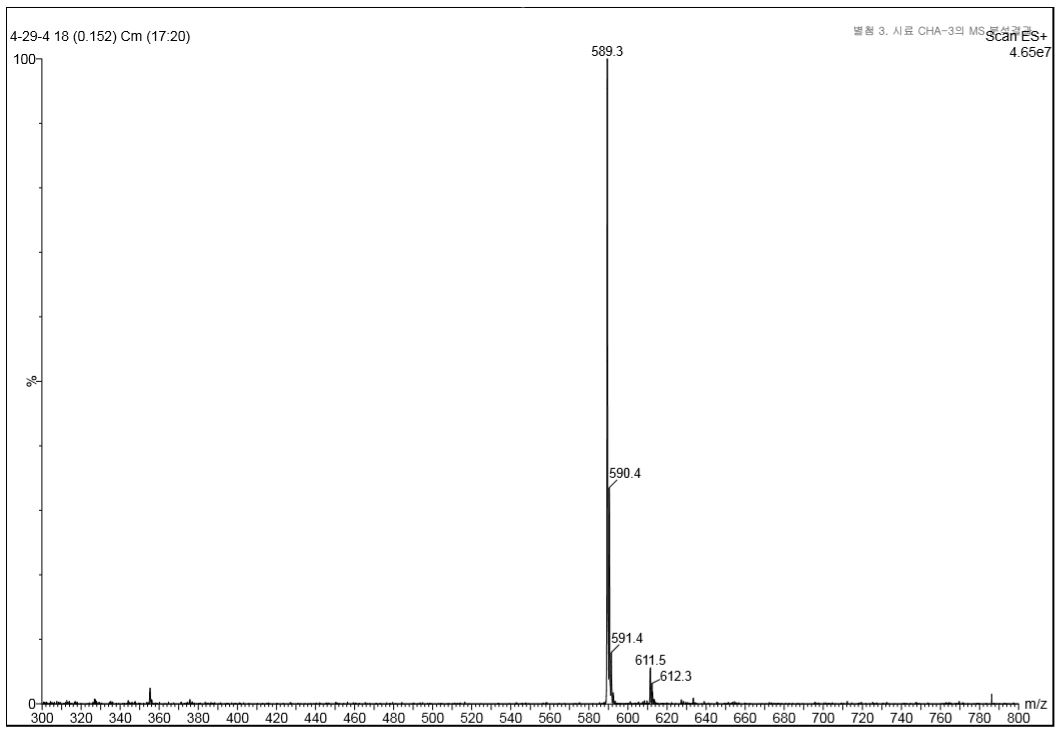


**Figure S2.** LC-Mass spectrum of cilengitide.


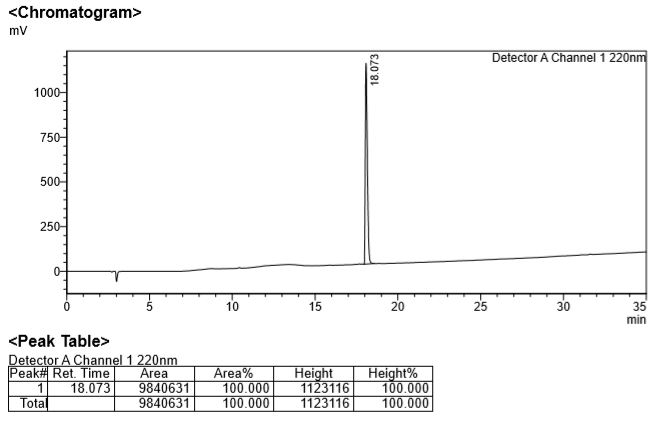


**Figure S3.** HPLC spectrum of cilengitide.


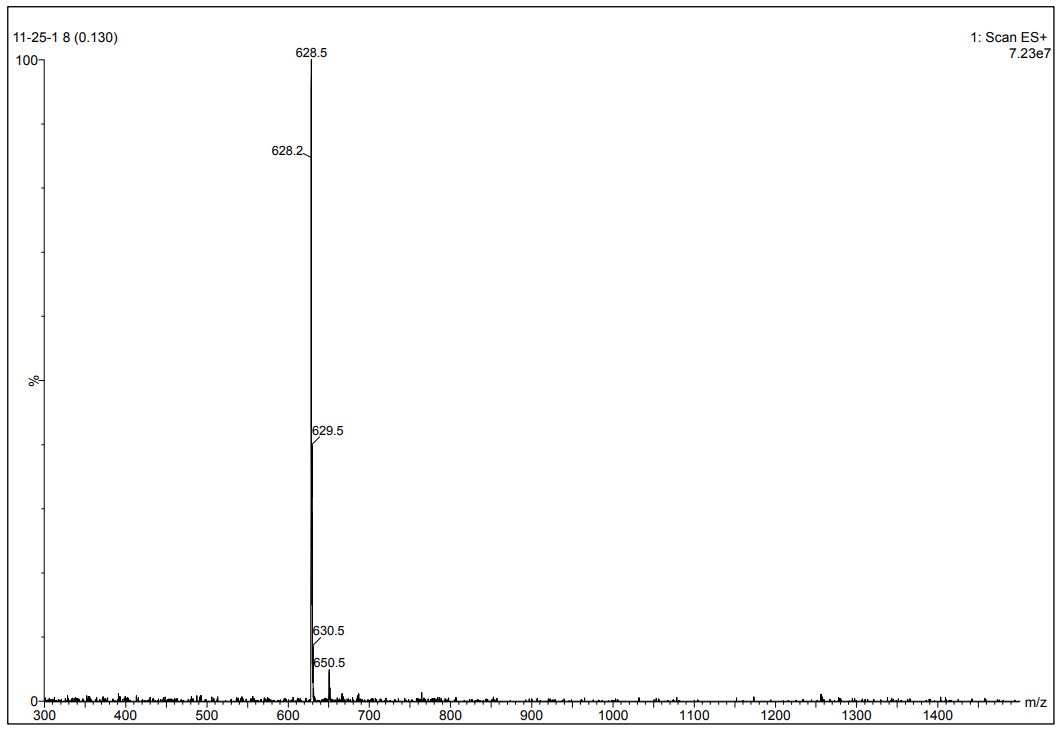


**Figure S4.** LC-Mass spectrum of compound R-1 (cRGDwV).

**
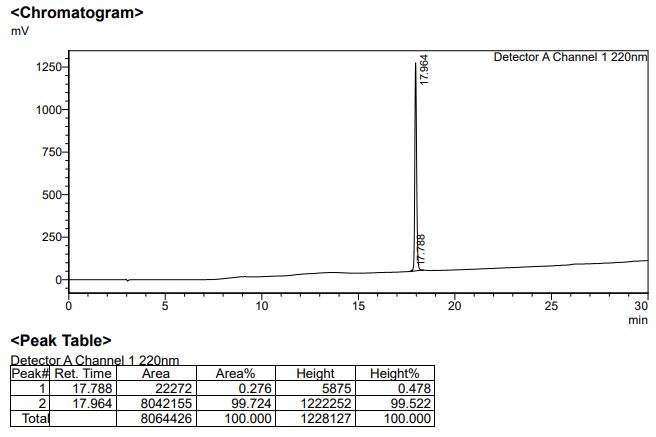
**

**Figure S5.** HPLC spectrum of compound R-1 (cRGDwV).


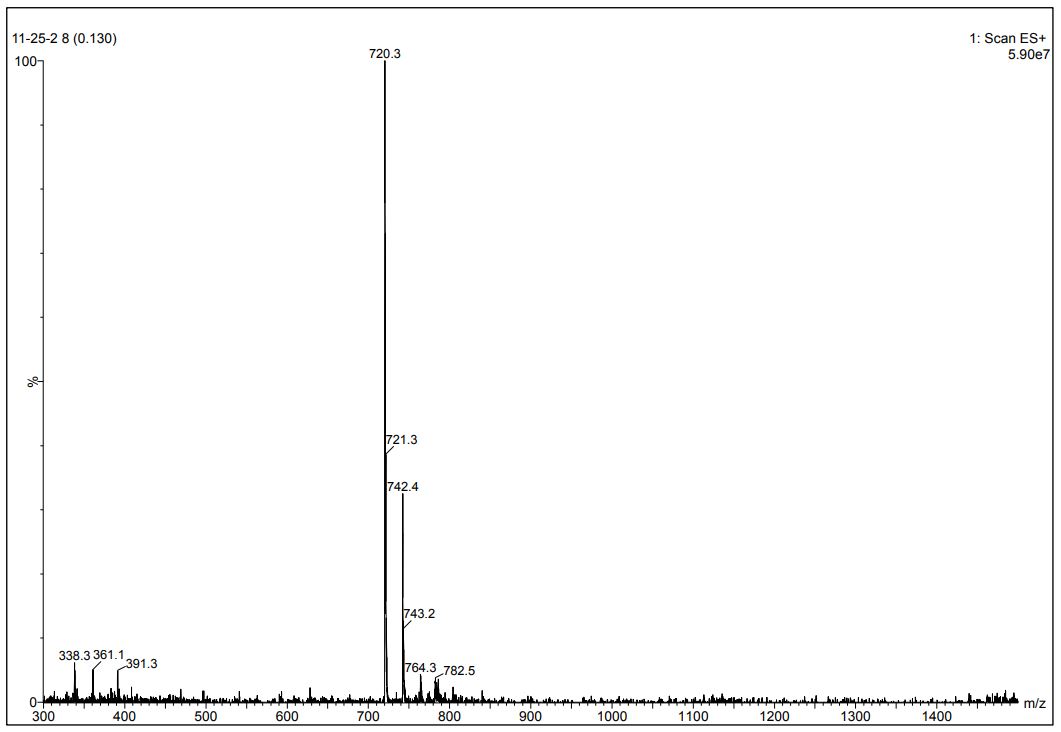


**Figure S6.** LC-Mass spectrum of compound R-2.


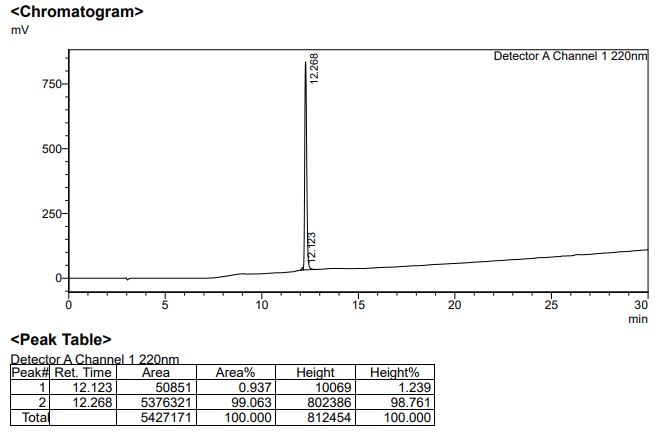


**Figure S7.** HPLC spectrum of compound R-2.


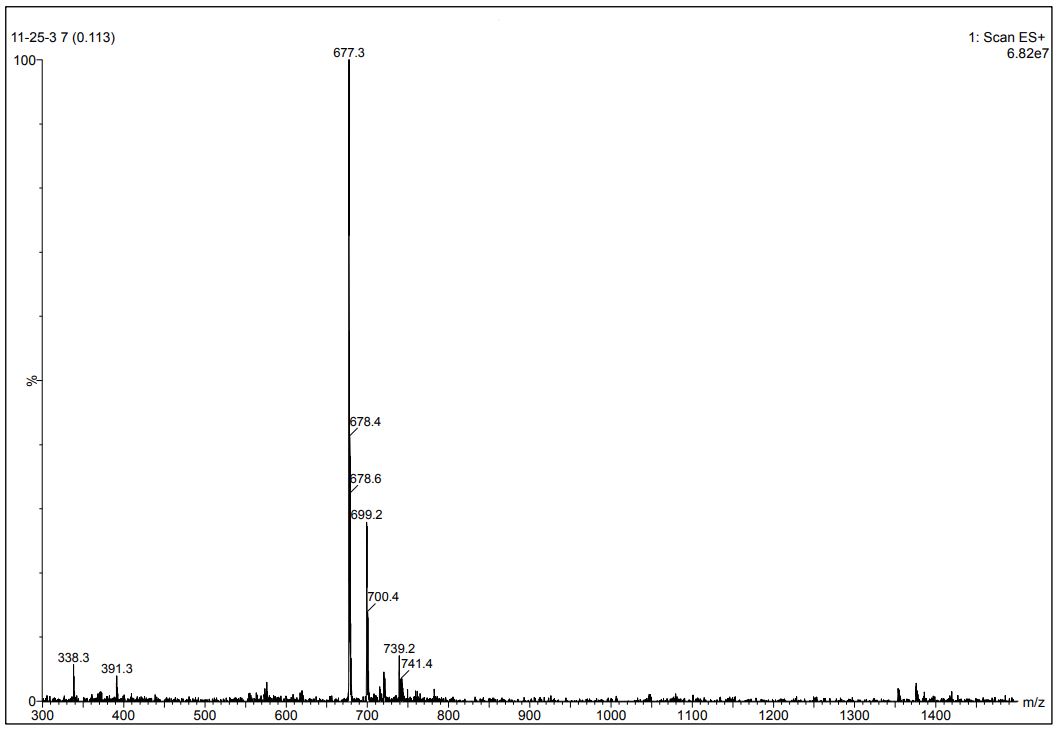


**Figure S8.** LC-Mass spectrum of compound R-3.

**
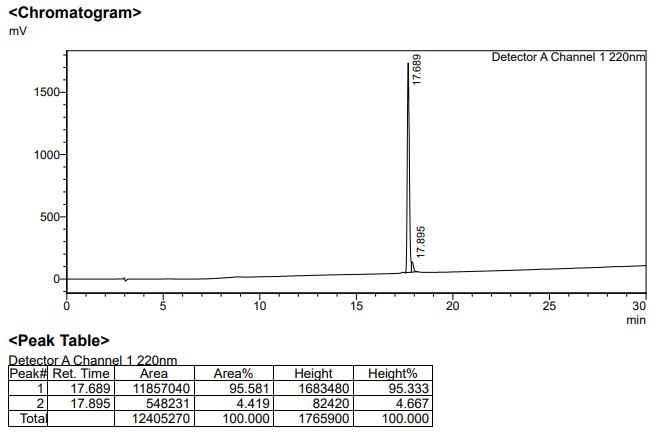
**

**Figure S9.** HPLC spectrum of compound R-3.


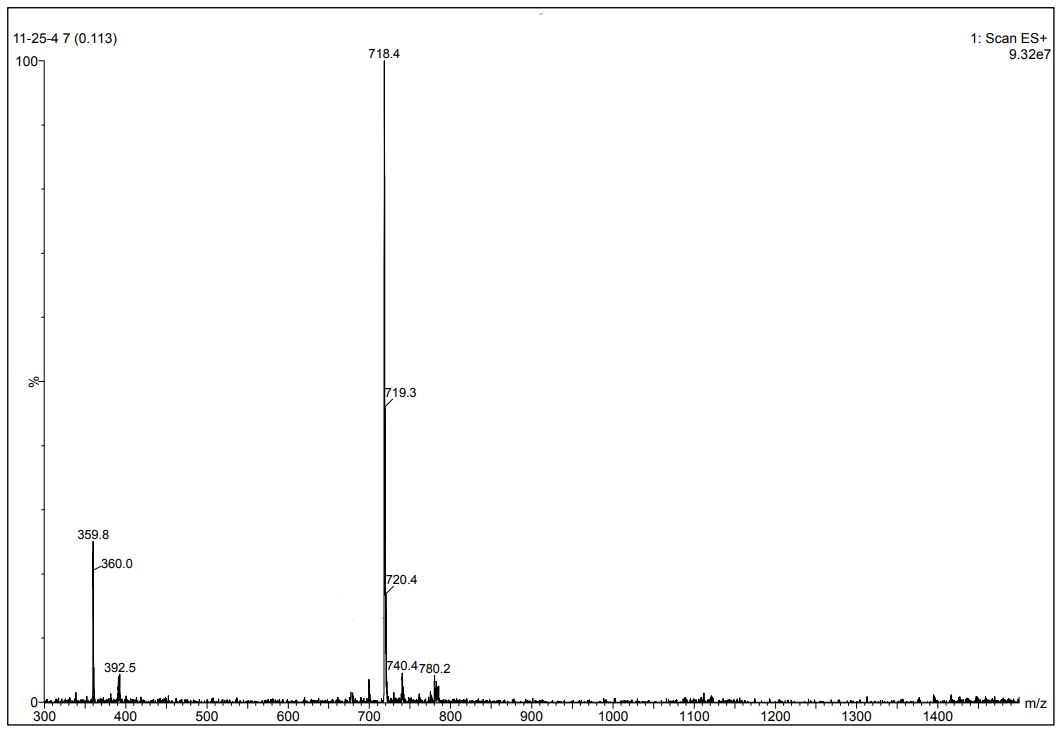


**Figure S10.** LC-Mass spectrum of compound R-4.


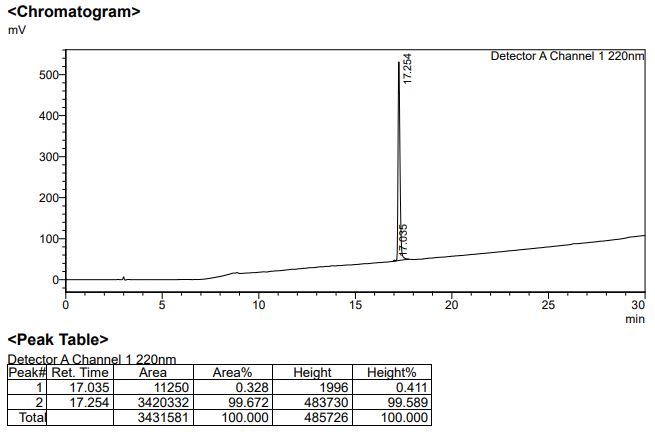


**Figure S11.** HPLC spectrum of compound R-4.


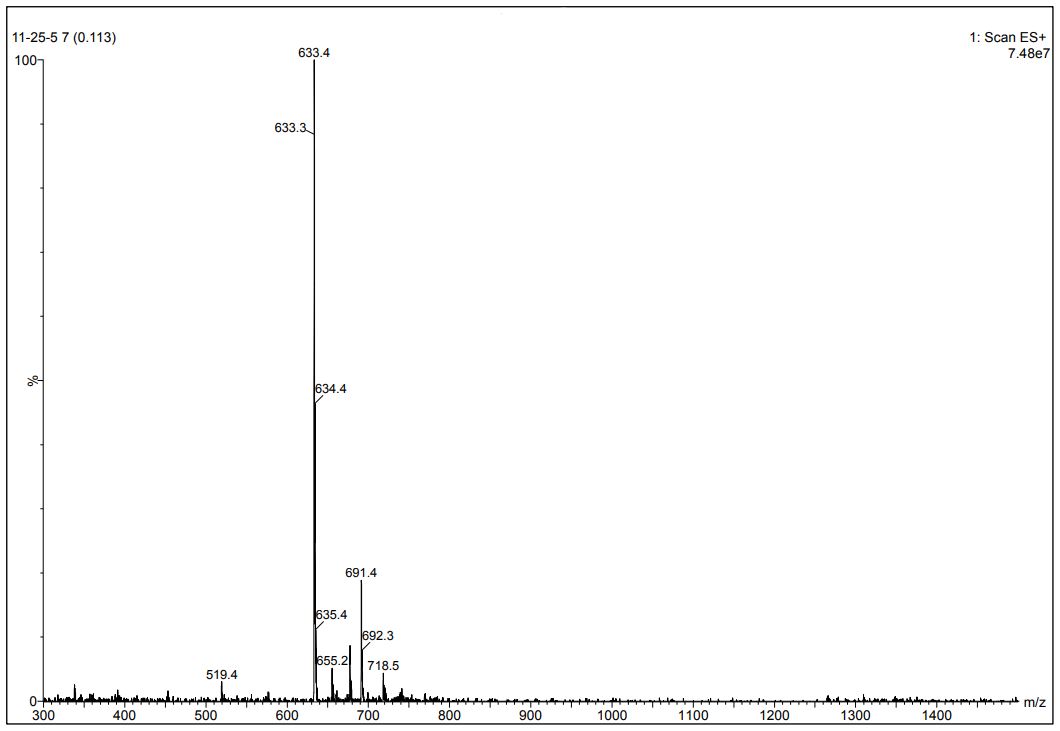


**Figure S12.** LC-Mass spectrum of compound R-5.


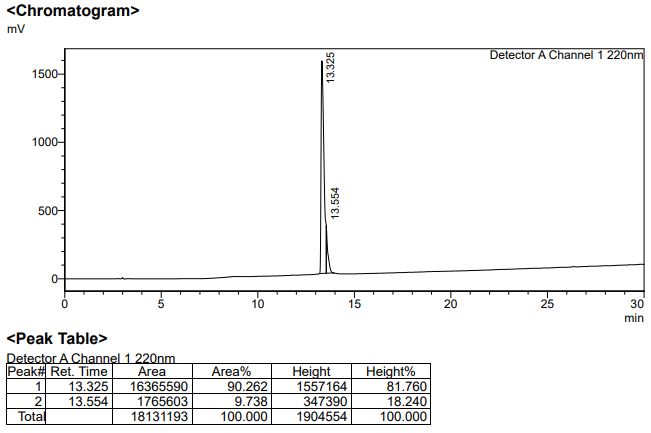


**Figure S13.** HPLC spectrum of compound R-5.


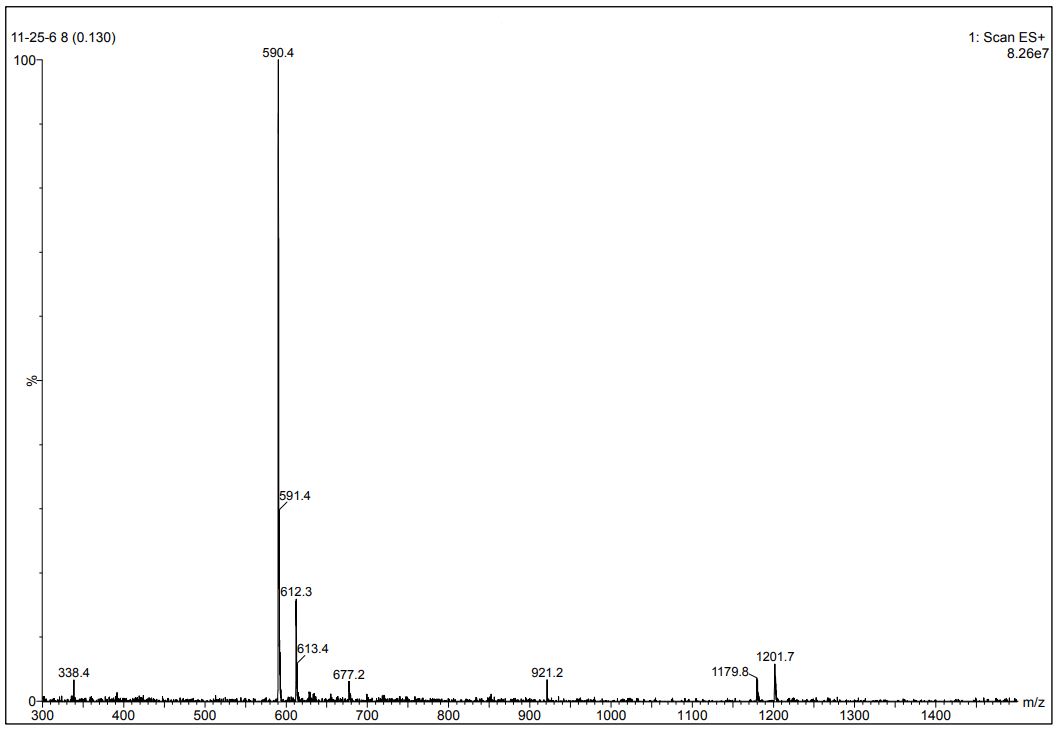


**Figure S14.** LC-Mass spectrum of compound R-6.


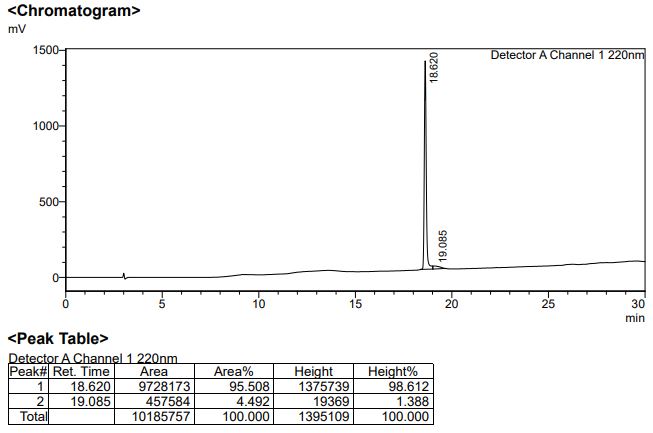


**Figure S15.** HPLC spectrum of compound R-6.


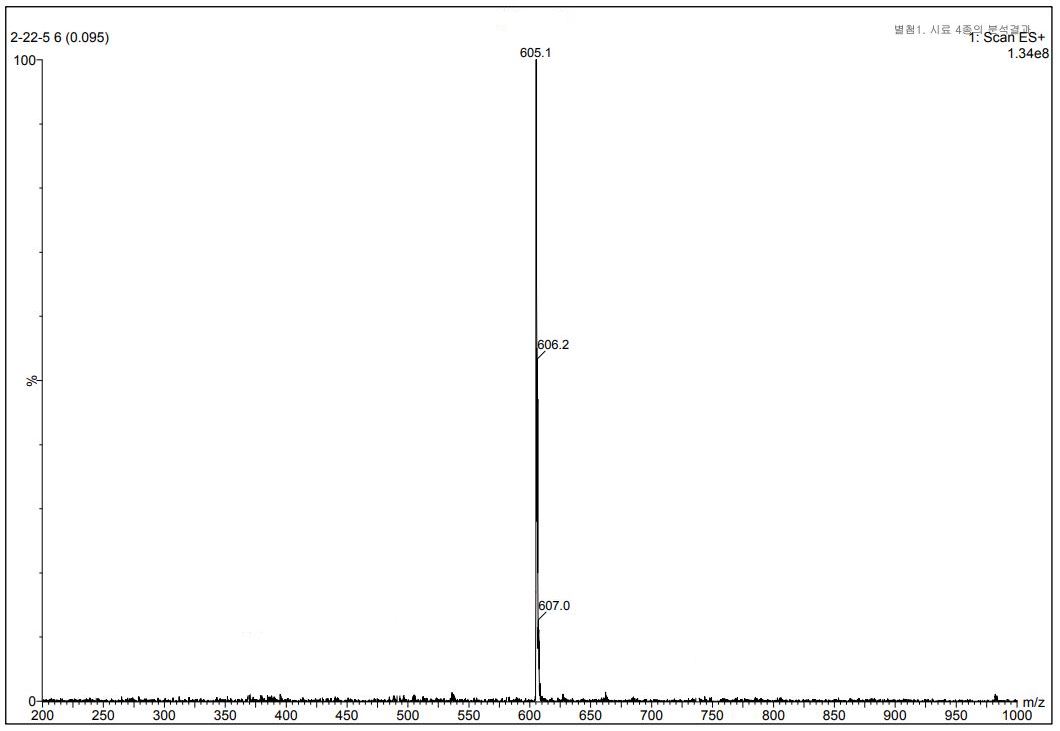


**Figure S16.** LC-Mass spectrum of compound R-7 (cRGDyV).


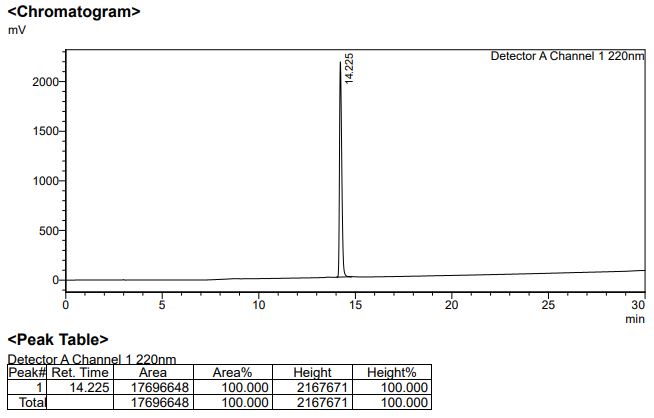


**Figure S17.** HPLC spectrum of compound R-7 (cRGDyV).


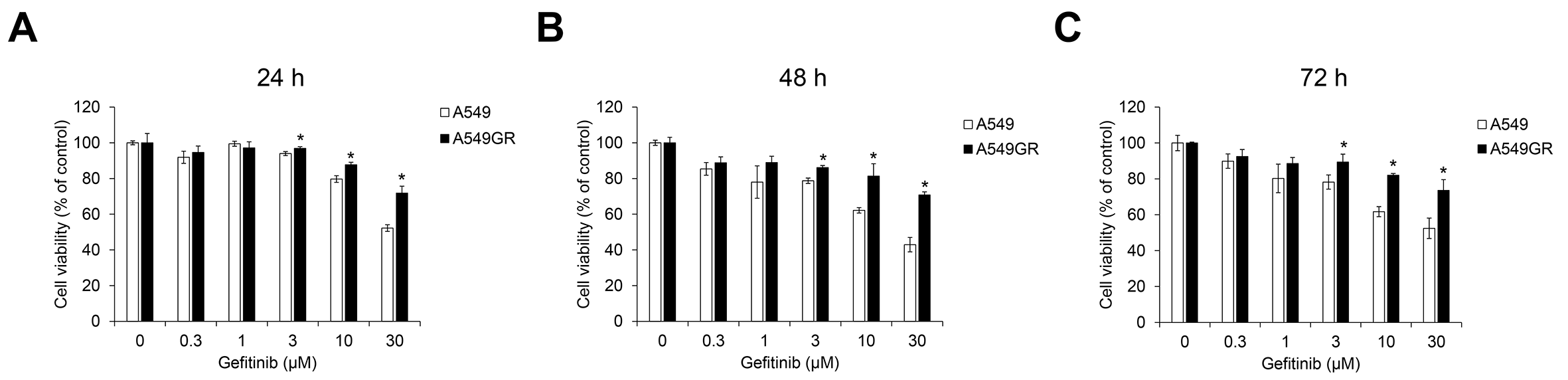


**Figure S18.** Effects on cell viability of gefitinib in wild-type A549 and gefitini-resistant A549 cells. (A-C) wild-type A549 (A549) and gefitinib-resistant A549 (A549GR) cells were treated with gefitinib for 24 h (A), 48 h (B), and 72 h (C). Cell viability was measured by the CCK-8 assay. Experiments were performed in triplicate. Data are mean ± SD. * *p* < 0.05 versus the cell viablity result of wild-type A549.

**
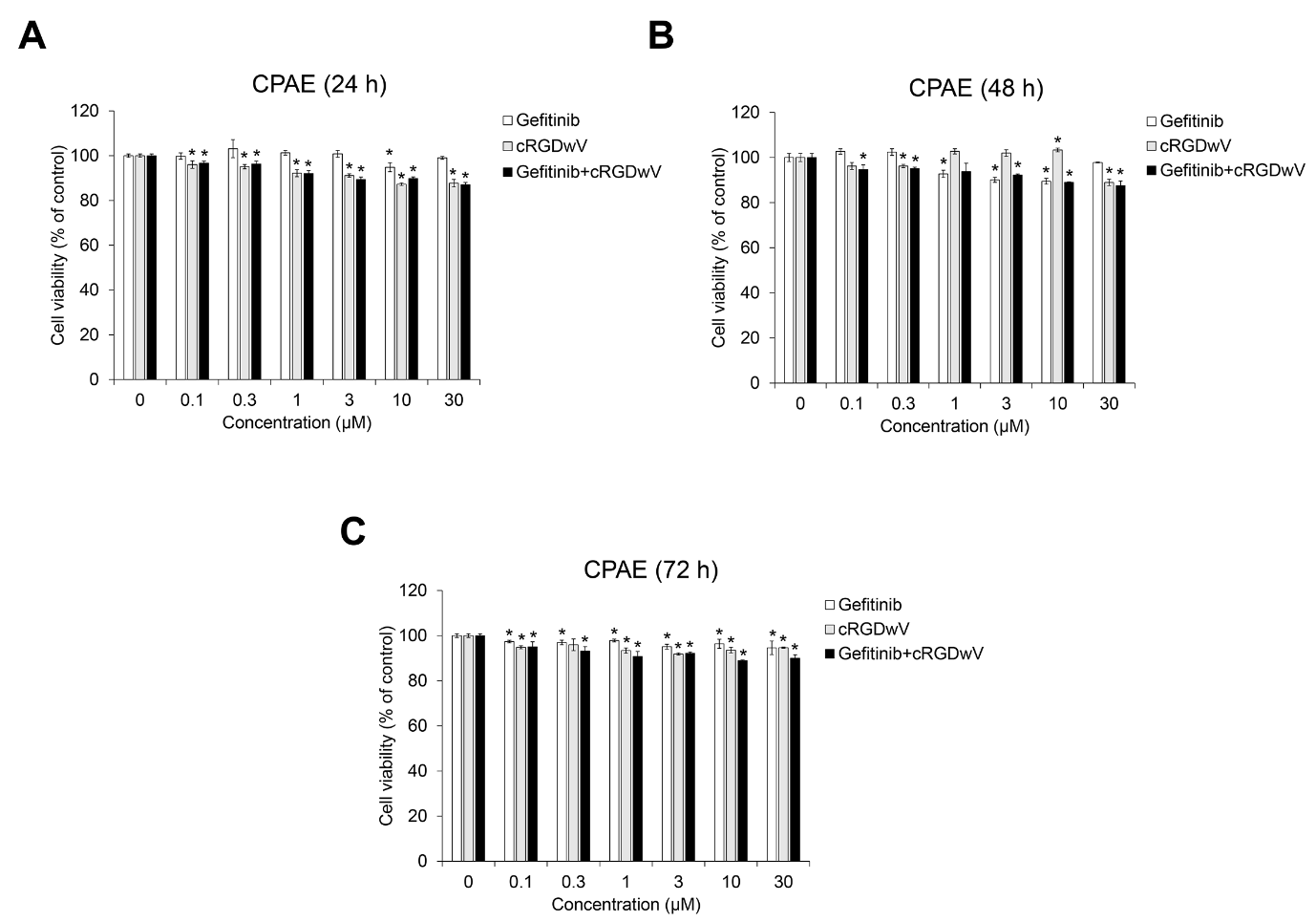
**

**Figure S19.** Effects on cell viability of gefitinib, cRGDwV, and combined treatment with gefitinib and cRGDwV in bovine lung endothelium CPAE cells. CPAE cells were treated with gefitinib or cRGDwV or a combination of gefitinib with cRGDwV for 24 h (A), 48 h (B), and 72 h (C). Cell viability was measured by the CCK-8 assay. Experiments were performed in triplicate. Data are mean ± SD. * *p* < 0.05 versus untreated control.


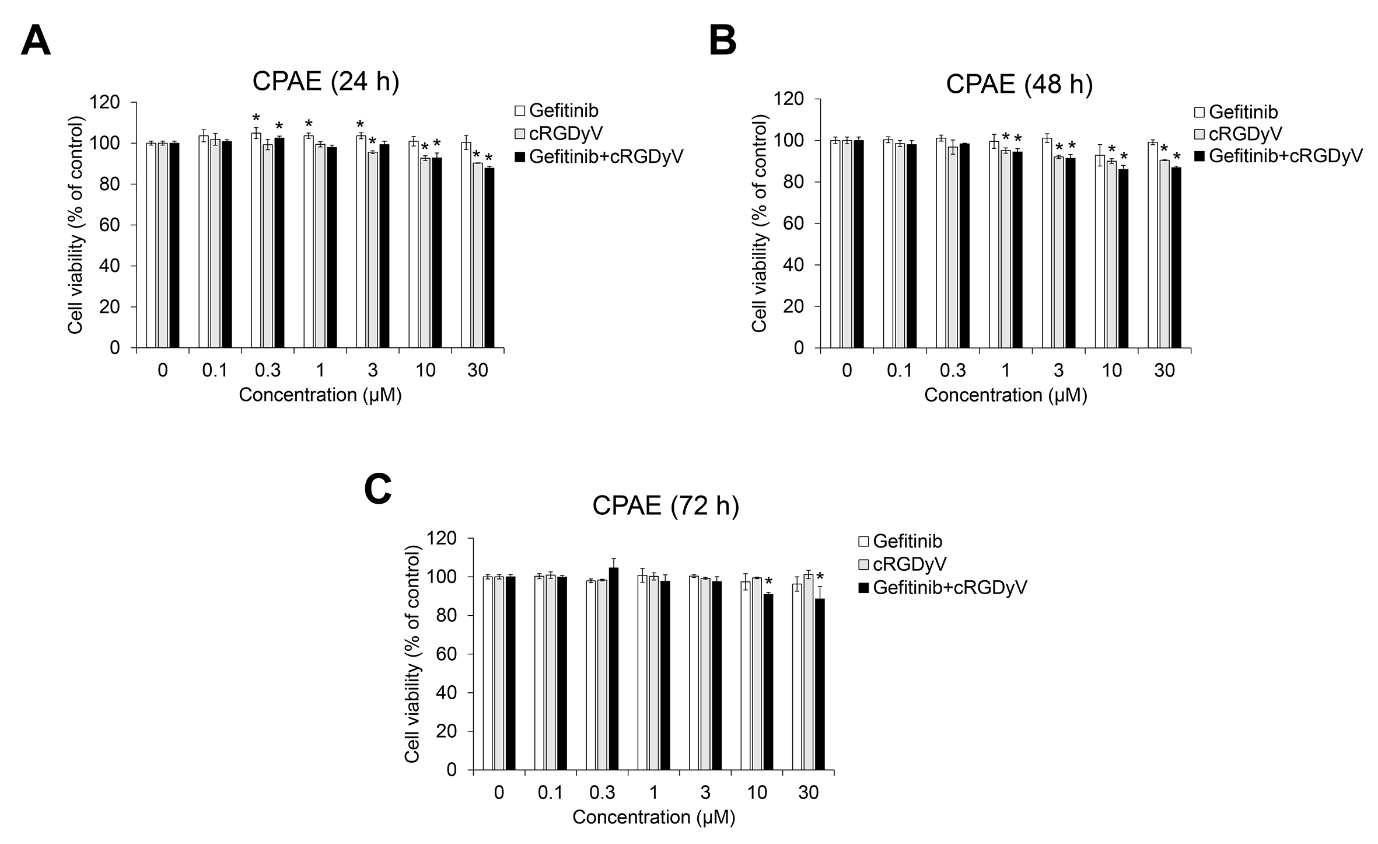


**Figure S20.** Effects on cell viability of gefitinib, cRGDyV, and combined treatment with gefitinib and cRGDyV in bovine lung endothelium CPAE cells. CPAE cells were treated with gefitinib or cRGDyV or a combination of gefitinib with cRGDyV for 24 h (A), 48 h (B), and 72 h (C). Cell viability was measured by the CCK-8 assay. Experiments were performed in triplicate. Data are mean ± SD. * *p* < 0.05 versus untreated control.

**
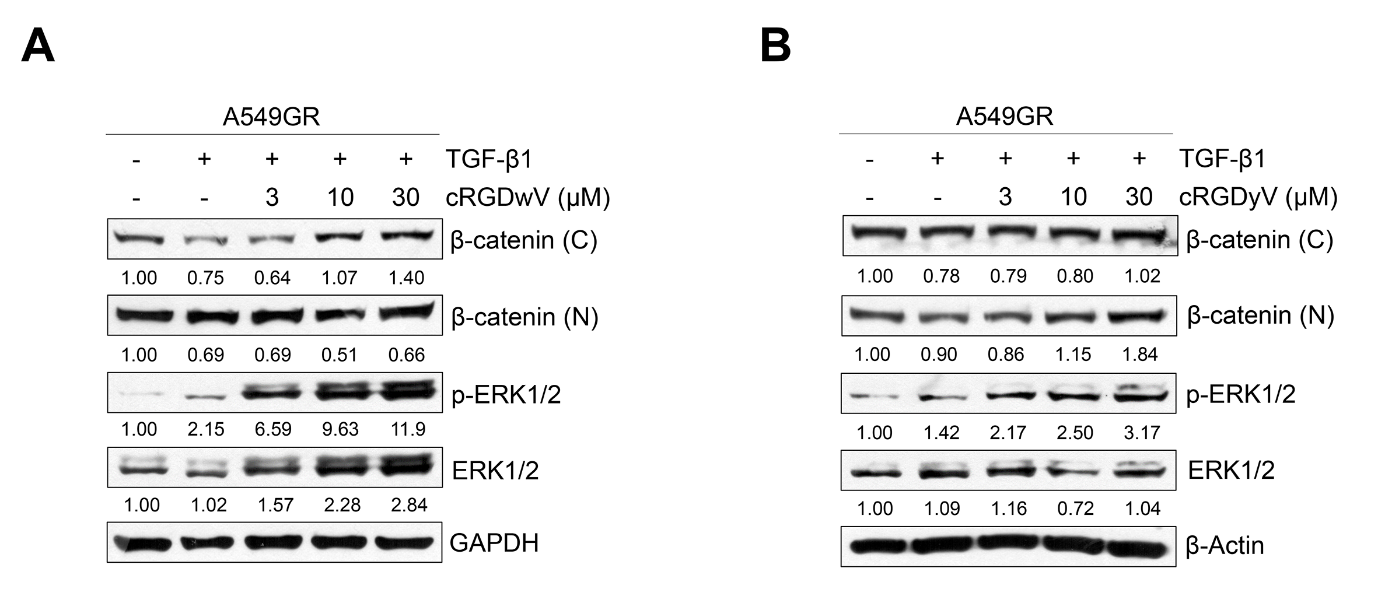
**

**Figure S21.** Effect of cilengitide derivatives on TGF-β1-induced expression of non-Smad signaling-related proteins in A549GR cells. Serum-deprived A549GR cells were treated with TGF-β1 (5 ng/mL) and cRGDwV (**A**) or cRGDyV (**B**) for 72 h. Expression of nuclear and cytosolic β-catenin and phosphorylation of ERK1/2 were determined by western blot analysis. GAPDH and β-actin were used as loading controls.

**
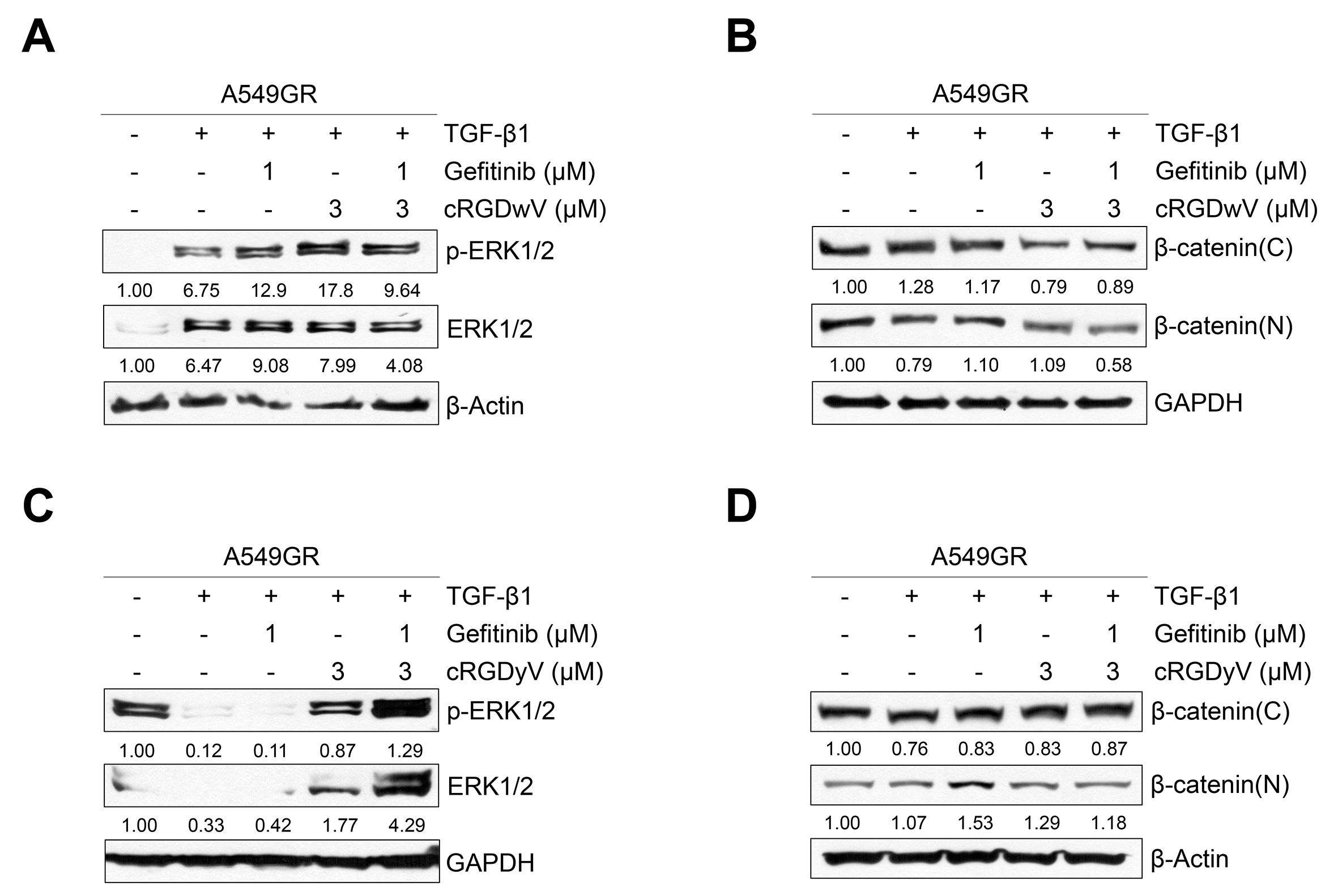
**

**Figure S22.** Combination effect of gefitinib with cilengitide derivatives on TGF-β1-induced expression of non-Smad signaling-related proteins in A549GR cells. (**A and B**) Serum-deprived A549GR cells treated with TGF-β1 (5 ng/mL) were treated with gefitinib (1 μM) or cRGDwV (3 μM) or with a combination of gefitinib and cRGDwV for 72 h. (**C and D**) Serum-deprived A549GR cells treated with TGF-β1 (5 ng/mL) were treated with gefitinib (1 μM) or cRGDyV (3 μM) or with a combination of gefitinib and cRGDyV for 72 h. Expression of nuclear and cytosolic β-catenin and phosphorylation of ERK1/2 was determined by Western blot analysis. GAPDH and β-actin were used as loading controls.

**Table S1.** Combination index (CI) values for the two-drug combination against A549GR cell viability.

| Incubation time (h) | Gefitinib (μM) | cRGDwV (μM) | CI value | cRGDyV (μM) | CI value |
| --- | --- | --- | --- | --- | --- |
| 24 | 0.1 | 0.1 | 0.5304 | 0.1 | 0.1514 |
|  | 0.3 | 0.3 | 0.1004 | 0.3 | 0.0338 |
|  | 1 | 1 | 0.6575 | 1 | 0.1111 |
|  | 3 | 3 | 0.1443 | 3 | 0.1308 |
|  | 10 | 10 | 0.1731 | 10 | 0.1459 |
|  | 30 | 30 | 0.0003 | 30 | 0.0095 |
| 48 | 0.1 | 0.1 | 0.3280 | 0.1 | 2.9573 |
|  | 0.3 | 0.3 | 0.3262 | 0.3 | 0.4979 |
|  | 1 | 1 | 0.5764 | 1 | 0.2808 |
|  | 3 | 3 | 0.2510 | 3 | 0.2170 |
|  | 10 | 10 | 0.0550 | 10 | 0.0315 |
|  | 30 | 30 | 0.0020 | 30 | 0.0000 |
| 72 | 0.1 | 0.1 | 0.0413 | 0.1 | 0.0010 |
|  | 0.3 | 0.3 | 0.1306 | 0.3 | 0.0033 |
|  | 1 | 1 | 0.4217 | 1 | 0.0278 |
|  | 3 | 3 | 1.0043 | 3 | 0.1213 |
|  | 10 | 10 | 0.3059 | 10 | 0.2805 |
|  | 30 | 30 | 0.0000 | 30 | 0.0001 |
